# Endoplasmic reticulum stress modulates the fate of lung resident mesenchymal stem cell to myofibroblast via C/EBP homologous protein during pulmonary fibrosis

**DOI:** 10.1186/s13287-022-02966-1

**Published:** 2022-06-28

**Authors:** Xiaoyu Yang, Wei Sun, Xiaoyan Jing, Qian Zhang, Hui Huang, Zuojun Xu

**Affiliations:** 1 Department of Respiratory and Critical Care Medicine, Peking Union Medical College Hospital, Chinese Academy of Medical Sciences and Peking Union Medical College, No. 1 Shuai Fu Yuan Street, Dong Cheng District, Beijing, 100730 People’s Republic of China; 2 Department of Respiratory and Critical Care Medicine, Sichuan Provincial People’s Hospital of Sichuan Academy of Medical Sciences, Chengdu, People’s Republic of China

**Keywords:** Lung resident mesenchymal stem cell, Endoplasmic reticulum stress, C/EBP homologous protein, Pulmonary fibrosis

## Abstract

**Background:**

As a fatal interstitial lung disease, idiopathic pulmonary fibrosis (IPF) was characterized by the insidious proliferation of extracellular matrix (ECM)-producing mesenchymal cells. Recent studies have demonstrated that lung resident mesenchymal/stromal cells (LR-MSC) are the source of myofibroblasts. Endoplasmic reticulum (ER) stress is prominent in IPF lung. This study sought to investigate the effects of ER stress on the behavior of LR-MSC during pulmonary fibrosis.

**Methods:**

ER stress and myofibroblast differentiation of LR-MSC in patients with IPF were evaluated. Primary mouse LR-MSC was harvested and used in vitro for testing the effects of ER stress and C/EBP homologous protein (CHOP) on LR-MSC. Adoptive transplantation of LR-MSC to bleomycin-induced pulmonary fibrosis was done to test the in vivo behavior of LR-MSC and its influence on pulmonary fibrosis.

**Results:**

We found that myofibroblast differentiation of LR-MSC is associated with ER stress in IPF and bleomycin-induced mouse fibrotic lung. Tunicamycin-induced ER stress impairs the paracrine, migration, and reparative function of mouse LR-MSC to injured type 2 alveolar epithelial cells MLE-12. Overexpression of the ER stress responder C/EBP homologous protein (CHOP) facilitates the TGFβ1-induced myofibroblast transformation of LR-MSC via boosting the TGFβ/SMAD signaling pathway. CHOP knockdown facilitates engraftment and inhibits the myofibroblast transformation of LR-MSC during bleomycin-induced pulmonary fibrosis, thus promoting the efficacy of adopted LR-MSC in alleviating pulmonary fibrosis.

**Conclusion:**

Our work revealed a novel role that ER stress involved in pulmonary fibrosis by influencing the fate of LR-MSC and transformed to “crime factor” myofibroblast, during which CHOP acts as the key modulator. These results indicate that pharmacies targeting CHOP or therapies based on CHOP knockdown LR-MSC may be promising ways to treat pulmonary fibrosis.

**Supplementary Information:**

The online version contains supplementary material available at 10.1186/s13287-022-02966-1.

## Introduction

Injured alveolar epithelial cells require repairment and regeneration, or they will be replaced by proliferated mesenchymal cells and their secreted extracellular matrix (ECM), leading to pulmonary fibrosis. At present, idiopathic pulmonary fibrosis (IPF) and other irreversible pulmonary fibrosis caused by various end-stage lung diseases are a challenge clinically because we have not fully understood the core mechanism of pulmonary fibrosis progression. One of the central points among these unknown issues is the cellular origin that produces the ECM during pulmonary fibrosis. With the development of lineage tracing techniques and single-cell transcriptomics, people gradually recognized that the pool of fibrogenic lung mesenchymal cells is a heterogeneous population with various origins [1]. Besides lung-resident fibroblasts and pericytes, a mesenchymal stem cell (MSC)-like group, lung resident mesenchymal stem cell (LR-MSC), was identified to maintain the differentiative capability for myofibroblasts, which act as the major source of ECM [2]. Evidence shows that LR-MSC also plays an essential role in maintaining local homeostasis in tissues under the physiological state [3, 4]. Like other tissue-derived MSCs, LR-MSC repair damaged epithelial cells by secreting growth factors [5], extracellular vesicles [6], and tight binding [7] during lung injury. However, the biological behavior and the role of endogenous LR-MSC during pulmonary fibrosis remain unclear.

Endoplasmic reticulum (ER) stress results from the dysfunction in ER in protein processing, leading to alterations in cell fate if the prolonged stress persists. Causes of this malfunction include inadequate cellular energy metabolism, genetic mutations that cause improper proteins fold, increased translation, and fewer chaperone molecules [8]. Under such conditions, cells undergo unfolded protein response (UPR) to relieve this stress, including reducing protein translation, upregulating chaperone molecules, altering the cell’s state, and inducing apoptosis if this does not work. A growing body of evidence demonstrated that ER stress was prominent in IPF, and multiple UPR-associated proteins overexpressed in IPF lungs [9–11]. Studies have shown that ER stress is involved in the pathological process of pulmonary fibrosis by affecting various cell types in the lung, including epithelial cells (inducing apoptosis and senescence) [9, 12], macrophages (switching to M2 polarization) [13], and fibroblasts (promoting fibrogenesis) [14]. However, the influence of ER stress on LR-MSC in pulmonary fibrosis remained unknown.

C/EBP homologous protein (CHOP) is a multifunctional transcription factor downstream of ER stress-induced UPR. Besides the major role in UPR-induced cell apoptosis, it also affects inflammatory response [15] and cell differentiation [16]. As for MSC, for example, CHOP was demonstrated to promote osteoblastic differentiation of murine stromal cells [17] and the odontoblastic potential of human dental pulp cells [18], both of which were MSC-like stromal progenitor cells. Up to date, there are no studies about the effects of CHOP on LR-MSC.

In this study, based on the extensive and important role of ER stress in pulmonary fibrosis and the potential impact on the biological behavior of MSCs, we hypothesized that ER stress promotes the transformation of LR-MSC into myofibroblasts, which are involved in the process of pulmonary fibrosis. The intervention of CHOP expression of LR-MSC may eliminate the harmful effects of LR-MSC and is promising in cell-based therapy for pulmonary fibrosis.

## Materials and methods

### Collection of human lung tissue

This study was approved by the Ethics Committee of Peking Union Medical College Hospital (JS-1127). IPF lung tissue samples (*n* = 6) were obtained from the department of lung transplantation, the First Affiliated Hospital of Guangzhou Medical University, Guangzhou, China. The IPF diagnose was made by the guidance of the 2018 ATS/ERS/JRS/ALAT guideline [19]. Non-fibrotic control lung tissues (*n* = 6) were obtained from patients with tumors from the Thoracic Surgery Department of Peking Union Medical College Hospital, Chinese Academy of Medical Sciences. Information about the donors for IPF and control lung tissue samples is listed in Additional file 7: Table E1. Each lung tissue sample was divided into two parts: One part was stored in liquid nitrogen for immunoblot analysis, and the other part was fixed with 4% neutral phosphate-buffered paraformaldehyde overnight, dehydrated, transparentized, and embedded in paraffin before being sectioned into 5-µm-thick slices for pathological and immunofluorescence staining.

### Animal care and a mouse model of pulmonary fibrosis

Animal experiments were approved by the Chinese Academy of Medical Sciences Laboratory Animal Center. All the operations were conducted in accordance with the regulations established by the Institutional Committee for the Care and Use of Laboratory Animals. Six- to 8-week-old male C57BL/6 mice (specific pathogen-free) were obtained from the Laboratory Animal Center, Peking Union Medical College Hospital, and housed at a constant room temperature with a 12-h light/dark cycle.

The mice were injected intratracheally with 50 µl of 5 mg/kg bleomycin. The control group received 50 µl saline injection intratracheally. On day 21, the mice were killed, and lungs were collected for subsequent experiments.

### Isolation of mouse LR-MSC

Six 4–6-week-old male C57BL/6 mice were selected for LR-MSC isolation each time. The finely minced lung tissues were immersed into HBSS (with Ca^2+^ and Mg^2+^) with 200U/mL Collagenase I (Gibco, Grand Island, NY, USA) and 2U/mL Dispase I (Sigma-Aldrich, St. Louis, USA) and digested for 1 h in the condition of 37 °C water bath with frequent agitation. Single-cell suspensions were harvested by passing through a 70-μm mesh filter and RBC lysis. LR-MSC was isolated by depleting CD31, CD45, and EpCam-positive cells and then collecting Sca1-positive cells by MACS (Miltenyi Biotec, Auburn, CA, USA)’s protocol.

### Cell culture and treatment

Isolated LR-MSC was cultured in DMEM medium (Corning, New York, USA) with 20% FBS (Gibco, Grand Island, NY, USA), 1% penicillin, and streptomycin, maintained in a humidified atmosphere of 5% CO_2_ at 37 °C. Cells were passaged 1:2 using TrypLE™ Express Enzyme (Gibco, Grand Island, NY, USA) when they reached 80–90% confluence. Mouse alveolar epithelial cell line MLE-12 was purchased from American Type Culture Collection (ATCC, Manassas, Virginia, USA). MLE-12 was maintained in high-glucose DMEM supplemented with 10% FBS, penicillin, and streptomycin in a humidified atmosphere of 5% CO_2_ at 37 °C. Tunicamycin was purchased from Sigma-Aldrich and TGFβ1 from Proteintech.

### Immunofluorescence staining

Lung tissue sections were deparaffinized with xylene for 20 min twice and rehydrated in gradient reduced ethanol (100% twice, 95%, 90%, 75%, and 50% once) for 5 min each. Antigen retrieval was performed for 30 min at 95 °C in 0.01 M pH 6.0 sodium citrate buffer. The sections were then blocked with 10% goat serum in PBST at room temperature for 1 h. For human lung tissue, the slides were incubated with primary antibodies as follows: rabbit anti-CD90 antibody (Cell Signaling, #13,801), mouse anti-alpha smooth muscle actin antibody (Abcam, ab119952), and mouse anti-CHOP (DDIT3) antibody (Abcam, ab11419). For mouse lung tissue, the primary antibodies were rabbit anti-Sca1 antibody (Abcam, ab109211), mouse anti-alpha smooth muscle actin antibody (Abcam, ab119952), mouse anti-CHOP (DDIT3) antibody (Abcam, ab11419), rabbit anti-Collagen I antibody (Abcam, ab138492), and rabbit anti-Grp78 antibody (Abcam, ab21685). Rabbit IgG (Abcam, ab172730) and mouse IgG (Abcam, ab37355) were isotypes for primary antibodies. They were then incubated overnight at 4 °C. The LR-MSC grown on the glass cover was fixed and permeabilized with 0.2% Triton X 100. After blocking, cells were incubated with primary antibodies specified to α-SMA and Collagen I. The secondary antibodies were Alexa Fluor 488-conjugated goat anti-rabbit IgG antibody (Jackson ImmunoResearch, Cambridge, UK) and Alexa Fluor 594-conjugated goat anti-mouse IgG antibody (Jackson ImmunoResearch, Cambridge, UK), incubated for 1 h at room temperature.

### Immunofluorescence image quantification

For quantification of protein expression of LR-MSC, 10 fields were randomly selected, and the fluorescence intensity for each RGB channel was analyzed with Fiji software. Cell counts were performed by automatically calculating for DAPI by Fiji software using the Analyze Particles tool. For the quantification of the number of Grp78 and α-SMA-positive GFP^+^ cells, we randomly selected 10 fields (200 ×) of view and manually counted them by two separate counters. At least three animals per group were used.

### Fluorescence-activated cell sorting (FACS)

Prepared single-cell suspensions of digested lung tissue or ex vivo cultured LR-MSC were incubated with fluorescently labeled antibodies for 30 min in the dark situation. Antibodies for identification of MSC characteristics include anti-Sca1-FITC (Miltenyi Biotec, Auburn, CA, USA), anti-CD31-APC, anti-CD45-APC (BD Pharmingen, Franklin Lakes, NJ, USA), anti-CD90.2-FITC (Proteintech, Rosemont, IL), and anti-CD105-FITC (Abcam, ab184667). Expression of cell surface marker of LR-MSC was analyzed with FACSCelesta cytometer (BD Biosciences, San Jose, CA, USA), and data were acquired with BD FACS Diva software. Data were analyzed by FlowJo V10 (Tristar). The LR-MSC in single-cell suspensions of the digested lung was gated by Sca1^+^CD45^−^CD31^−^ and depleted EpCam^+^ epithelial cells using anti-EpCam-PE (Abcam, ab237387). The instilled GFP^+^ LR-MSC was sorted using BD FACSAria II flow cytometry for qPCR analysis.

### Induction of LR-MSC for multilineage differentiation

LR-MSC was seeded in 6-well plates (coat plates with 0.1% gelatin prior to osteogenic differentiation). Medium for C57BL/6 MSC differentiation induction was purchased from Cyagen Biosciences. After induction, the adipocytes, osteocytes, and chondrocytes were stained by oil red O, Alizarin red, and Alcian blue.

### Western blot

Total proteins were collected by cell lysis with RIPA buffer with proteinase inhibitor on ice. Nuclear protein was prepared with cytoplasmic and nuclear extraction kits (Invent Biotechnologies, Eden Prairie, MN, USA) following the manufacturer’s instructions. Protein samples were separated by 10% SDS-PAGE and transferred to a polyvinylidene fluoride (PVDF) membrane and blotted with specific antibodies.

### Primary antibodies for western blot

Primary antibodies for IPF lung tissue include ATF6 (Abcam, ab37149), PERK (Abcam, ab229912), IRE1 (Abcam, ab37073), Grp78 (Abcam, ab21685), CHOP (Abcam, ab11419), α-SMA (Abcam, ab119952), and Collagen I antibody (Abcam, ab138492). For mouse LR-MSC, they include ATF6 (Abcam, ab37149), PERK (Abcam, ab229912), IRE1 (Abcam, ab37073), Grp78 (Abcam, ab21685), CHOP (Abcam, ab11419), α-SMA (Abcam, ab119952), Collagen I antibody (Abcam, ab21286), anti-Smad2/3 (Abcam, ab202445), anti-pSmad2 (Abcam, ab188334), anti-pSmad3 (Abcam, ab529035), anti-Wnt10a (Abcam, ab106522), and anti-beta Catenin (Abcam, ab32572). Anti-Lamin B1 (Abcam, ab16048) was used for loading control of nuclear protein. Anti-GAPDH (Abcam, ab8245) was used for loading control for total protein.

### Analysis of western blot

The immunoreactive protein bands were detected using a chemiluminescence device (GE, Amersham Imager 680). The protein expression levels were evaluated by measuring the gray value of the band by Fiji software, *n* = 3 in each group, and the relative value to β-actin was compared.

### MTT assay

LR-MSC (2.5 × 10^5^ cells/mL) were seeded into a 96-well plate. Then, cells were maintained in DMEM with or without tunicamycin in the incubator at 5% CO_2_ and 37 °C. The plate was removed on 24 h and 48 h for the MTT assay. Briefly, 10µL MTT solution (5 g/L) (Solarbio, Beijing, China) was added to each well. Cells were then further incubated at 37 °C for 24 h. This was followed by the addition of 100μL DMSO. The plate was then slightly shaken and mixed evenly for 10 min. An automatic enzyme-labeled reading meter (Thermo) was used to measure the optical density value at 490 nm.

### Apoptosis detection

The adherent cells were digested with trypsin (EDTA free) and incubated with Annexin V-FITC and cell death dye 7-AAD. Then, the apoptotic LR-MSC or MLE-12 was evaluated by the percentage of Annexin V-positive cells using FACS.

### Enzyme-linked immunosorbent assay

Media of cocultured LR-MSC and MLE-12 was collected 48 h after inoculation. The concentration of KGF, HGF, and TGFβ1 in the media was determined using KGF kit (R&D Systems), mouse HGF ELISA kit (Abcam, ab223862), and mouse TGF beta 1 ELISA kit (Abcam, ab119557) according to manufacturer’s instructions.

### Transwell migration assay

Transwell migration assay was performed using Millipore transwell chambers (8 μm pore size, Millipore). LR-MSC (2 × 10^4^ in each well) were seeded in the upper chambers of the 24-well plate (Corning, New York, USA) in 100 μL serum-free medium. MLE-12 (2 × 10^4^ in each well) were seeded in the lower chambers in 600 μL complete medium. 5 μg/mL bleomycin or 100 ng/mL tunicamycin concentration of the medium is added if necessary. The chamber was incubated at 37 °C for 24 h. At the end of incubation, the cells in the upper surface of the membrane were removed with a cotton swab. Cells in the lower chamber were fixed with methanol and stained with Giemsa (HiMedia Labs). The images were taken with an inverted microscope (CX41, Olympus) and analyzed using NIH Fiji software.

### Cell transfection

Lentivirus-packaged CHOP-specific shRNA and overexpression plasmid were purchased from GenePharma for ex vivo and in vivo experiments, respectively. Negative scrambled control senseThe shRNA sequence used for negative scrambled controls was 5′-UUCUCCGAACGUGUCACGUTT-3′ (sense), and 5′-ACGUGACACGUUCGGAGAATT-3′ (antisense). The shRNA sequence for CHOPshRNA was 5′-UCGCUCUCCAGAUUCCAGUTT-3′ (sense), and 5′-ACUGGAAUCUGGAGAGCGATT-3′ (antisense). CHOP-knockdown LR-MSC for bronchial installation were constructed by infection of lentivirus containing CHOPshRNA. After infecting LR-MSC for 48 h, cells were purified by puromycin. SMAD luciferase reporter was purchased from Genomeditech (Shanghai, China), where multiple SMAD-responsive element was inserted before the luciferase gene.

### Immunoprecipitation

The solution and reagents for immunoprecipitation were purchased from Cell Signaling Technology. Cell lysates of harvested LR-MSC were pre-cleared by incubating with protein G magnetic beads to reduce the nonspecific binding. Then, pre-cleared cell lysates were incubated with primary antibodies with overnight rotation at 4 °C. Primary antibody for immunoprecipitation of C/EBPβ and CBP includes anti-CEBP beta antibody (Abcam, ab32358), anti-CREBBP antibody (Abcam, ab10489), and rabbit mAb IgG XP® Isotype Control (Cell Signaling Technology, #3900). The immunocomplex was added to pre-washed protein G magnetic beads and incubated for 20 min at room temperature. Pellet beads using magnetic separation rack then resuspend beads pellet and heat to 100 °C for disassociation. After removing the beads, supernatants were used for Western blotting.

### Treatment of mice with LR-MSC

The exogenous LR-MSC or LR-MSC-shCHOP (2 × 10^5^ in 100μL PBS) were given intratracheally once at the same time to bleomycin administration (day 0). Twenty-one days later, mice were euthanized for hydroxyproline assay, histology staining, and immunofluorescence, to evaluate the severity of pulmonary fibrosis and changes in adopted LR-MSC.

### Hydroxyproline assay

The whole lung was homogenized in ultra-pure water. The concentration of hydroxyproline was measured using Hydroxyproline Assay Kit (Colorimetric) (Abcam, ab222941) according to the manufacturer's protocol.

### Histology staining

Hematoxylin and eosin (HE) and Masson’s trichrome staining were performed on paraffin-embedded tissue sections. Ashcroft score was performed by averaging the scores of 10 random fields from one blinded and one non-blinded scorer. The fibrotic areas in lung tissue were evaluated using Fiji software using the collagen volume fraction.

### LR-MSC tracing in the lung

The intratracheally administrated LR-MSC was traced and quantified by fluorescence intensity of GFP in the lung by IVIS lumina II. At 1 and 21 days after LR-MSC treatment, lungs from BLM mice were harvested to evaluate the amount of exogenous LR-MSC within the lung.

### qPCR

mRNA level of genes in LR-MSC or lung tissue was quantitated by real-time PCR system, and results were analyzed after 40 cycles of amplification using ABI 7500 fast real-time PCR system. Relative expression levels of genes were defined by ∆∆Ct method and normalizing to control. Primer sequences are listed in Additional file 8: Table E2.

### Statistical analysis

Data were expressed as the mean ± SE. Statistical significance between two groups was estimated using the unpaired two-tailed Student’s *t* test. One-way ANOVA and post hoc Tukey’s test were performed for comparing more than two groups. *P* value < 0.05 was considered significant. All statistical analyses were performed using GraphPad Prism 9.0.

## Results

### LR-MSC in IPF patients undergo myofibroblast differentiation and ER stress-associated UPR

**Fig. 1:**
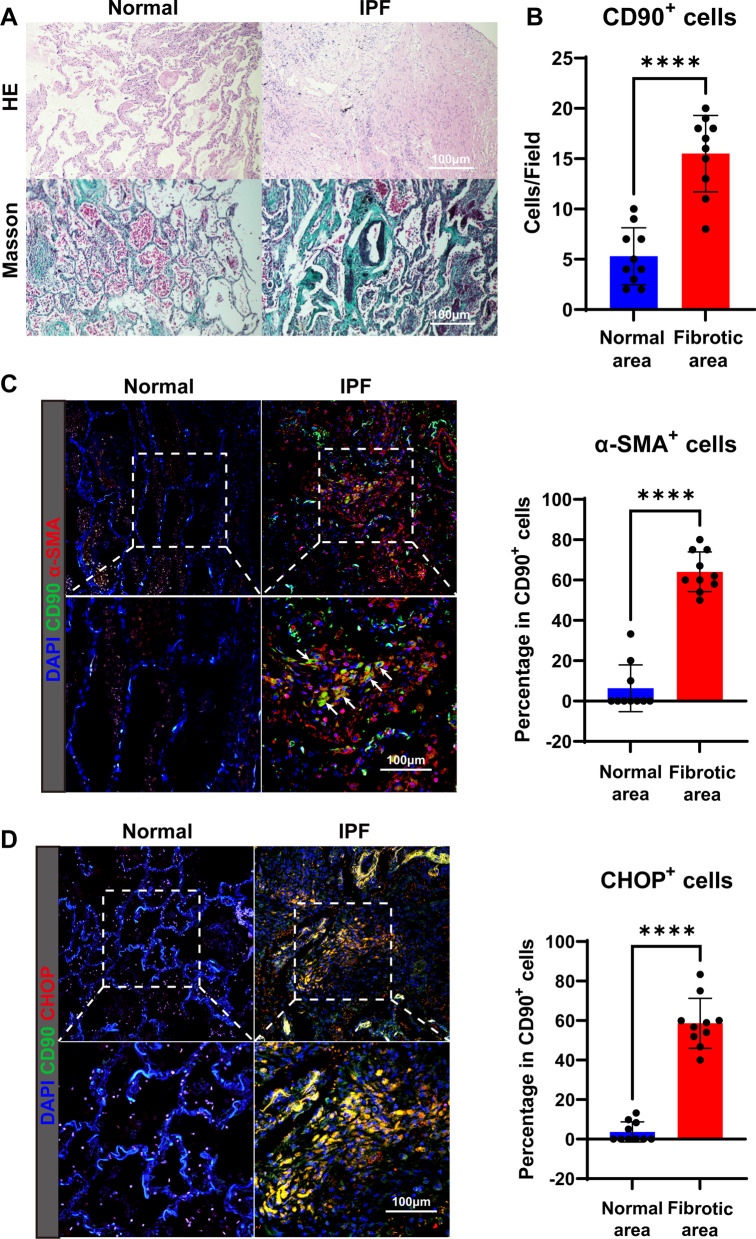
LR-MSC in IPF transformed to myofibroblast and suffered from ER stress. **A** HE and Masson’s staining of IPF lung show extensive fibroblastic foci and accumulation of extracellular matrix. **B** Quantification of CD90-positive cells in the normal or fibrotic area of IPF lung in each field. **C** Immunofluorescence of IPF and non-fibrotic (normal) lungs show the colocalization of LR-MSC marker CD90 and myofibroblast marker α-SMA. Arrows indicate positive staining of α-SMA in LR-MSC. The right panel shows the comparison between the percentage of α-SMA-positive CD90^+^ cells. **D** Immunofluorescence of IPF and non-fibrotic (normal) lungs shows CHOP expression in LR-MSC. The right panel shows the comparison between the percentage of CHOP-positive CD90^+^ cells

As reported in previous studies, LR-MSC in the human lung can be labeled by CD90 [20]. In this study, we collected lung tissues from IPF patients to trace the behavior of endogenous LR-MSC in the diseased setting. Pathological findings of IPF lung tissue show distortion of alveoli, thickened alveolar septum, formation of fibroblastic foci, and deposition of massive collagen (Fig. 1A). Immunofluorescence of lung tissues showed that the distribution of CD90-positive cells in the non-fibrotic lung was sparse and scattered in the normal lung, whereas the distribution is very concentrated in the fibroblastic foci of the IPF lung (Fig. 1B). We also found that a proportion of CD90-labeled cells of IPF patients expressed myofibroblast marker α-SMA (Fig. 1C) and aggregated in fibroblastic foci, which suggested that LR-MSC was the origin of the myofibroblast during fibrotic lung disease. We also detected excessive expression of UPR-related proteins, including ATF6, IRE1, PERK, Grp78, and the downstream transcript factor CHOP, reflecting widespread ER stress in the IPF lung (Additional file 1: Figure E1). To elucidate the role of ER stress in LR-MSC differentiation, we evaluated the expression of UPR-specific marker CHOP in LR-MSC. In normal lung, the distribution of LR-MSC in the alveolar interstitium was sparse without significant CHOP expression (Fig. 1D), while in IPF lung, LR-MSC in fibrotic foci showed a higher CHOP level, which indicated that the LR-MSC suffered from ER stress during pulmonary fibrosis.

### LR-MSC suffers from ER stress and myofibroblast differentiation during bleomycin-induced mouse lung fibrosis

**Fig. 2:**
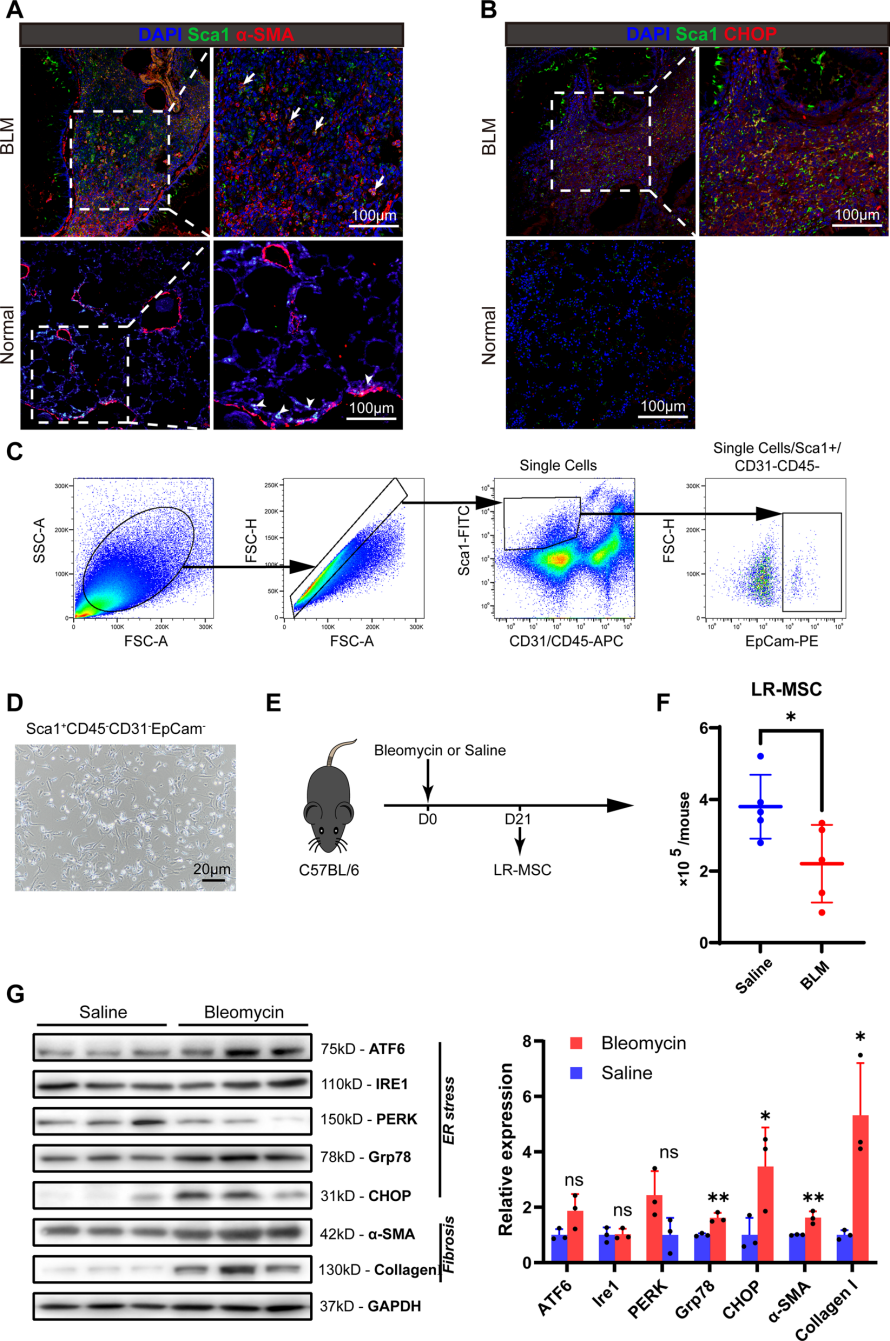
LR-MSC in bleomycin-induced pulmonary fibrosis (BLM) mirrors the behavior of which in IPF. **A** Immunofluorescence of BLM and normal lungs show the colocalization of mouse LR-MSC marker Sca1 and myofibroblast marker α-SMA. Arrows indicate positive staining of α-SMA in LR-MSC. Arrowheads indicate the parabronchial distribution of LR-MSC during homeostasis. **B** Immunofluorescence of BLM and normal lungs show the expression of CHOP in LR-MSC. **C** Gating strategy for analyzing the expression of EpCam in Sca1^+^CD45^−^CD31^−^ cells by FACS. **D** Isolated LR-MSC morphology was revealed by conventional light microscopy after 3 days of culture. **E** Schematic of the workflow used to isolate LR-MSC during homeostasis (Saline) and fibrosis (Bleomycin) for subsequent experiments. **F** Counts of isolated LR-MSC in each normal or BLM C57BL/6 mouse. **G** Western blot shows the expression of ER stress and fibrosis-associated proteins in isolated LR-MSC

We established a bleomycin-induced mouse model of pulmonary fibrosis (BLM) to track the behavior of LR-MSC during fibrosis. In normal conditions, LR-MSC (Sca1^+^) does not express α-SMA and locates in the parabronchial or perivascular mesenchyme (Fig. 2A), while in BLM lung, LR-MSC accumulates in the fibrotic area and expresses α-SMA. We then evaluated the effect of ER stress on LR-MSC in bleomycin-treated mice. The LR-MSC was distributed in fibrotic areas and with a higher level of CHOP (Fig. 2B). Thus, the clinical phenomenon in IPF was reproduced in the mouse model by the expression of α-SMA and CHOP in Sca1-labeled mouse LR-MSC.

Lung stem-like cells with the phenotype of Sca1^+^CD45^−^CD31^−^ were considered LR-MSC in mice [21, 22]. However, we found that about 8% of these cells were positive for EpCam, a surface marker for epithelial lineage (Fig. 2C). Thus, we isolated LR-MSC with the modified panel of Sca1^+^EpCam^−^CD45^−^CD31^−^ by magnetic-activated cell sorting (MACS) to conduct ex vivo experiments (Fig. 2C). When cultured in dishes, isolated LR-MSC adhered to the surface with stellate morphology like MSC from other origins (Fig. 2D). These cells neither express hematopoietic marker CD45, CD34, nor endothelial marker CD31, but they express CD90 and CD105 (Additional file 2: Figure E2A), consistent with MSC characteristics. We also tested their multipotent potential by inducing mesenchymal differentiation in vitro, including adipocyte, osteocyte, and chondrocyte (Additional file 2: Figure E2B). Thus, these extracted cells met the criteria for LR-MSC.

Abnormalities in LR-MSC were identified in bleomycin-treated mice (Fig. 2E). Firstly, the count of LR-MSC reduced dramatically (about 2 × 10^5^ per mouse) to half of that in normal conditions (Fig. 2F). Then, LR-MSC from model mice expresses a higher level of UPR-associated proteins such as ATF6, Grp78, and CHOP than the control group (Fig. 2G). As expected, myofibroblast marker α-SMA and fibrogenesis marker Collagen I also overexpressed. This phenomenon is consistent with that of IPF patients. However, a cause-and-effect relationship between ER stress and myofibroblast differentiation remains unknown.

### ER stress dampens the epithelium repair function of LR-MSC

**Fig. 3:**
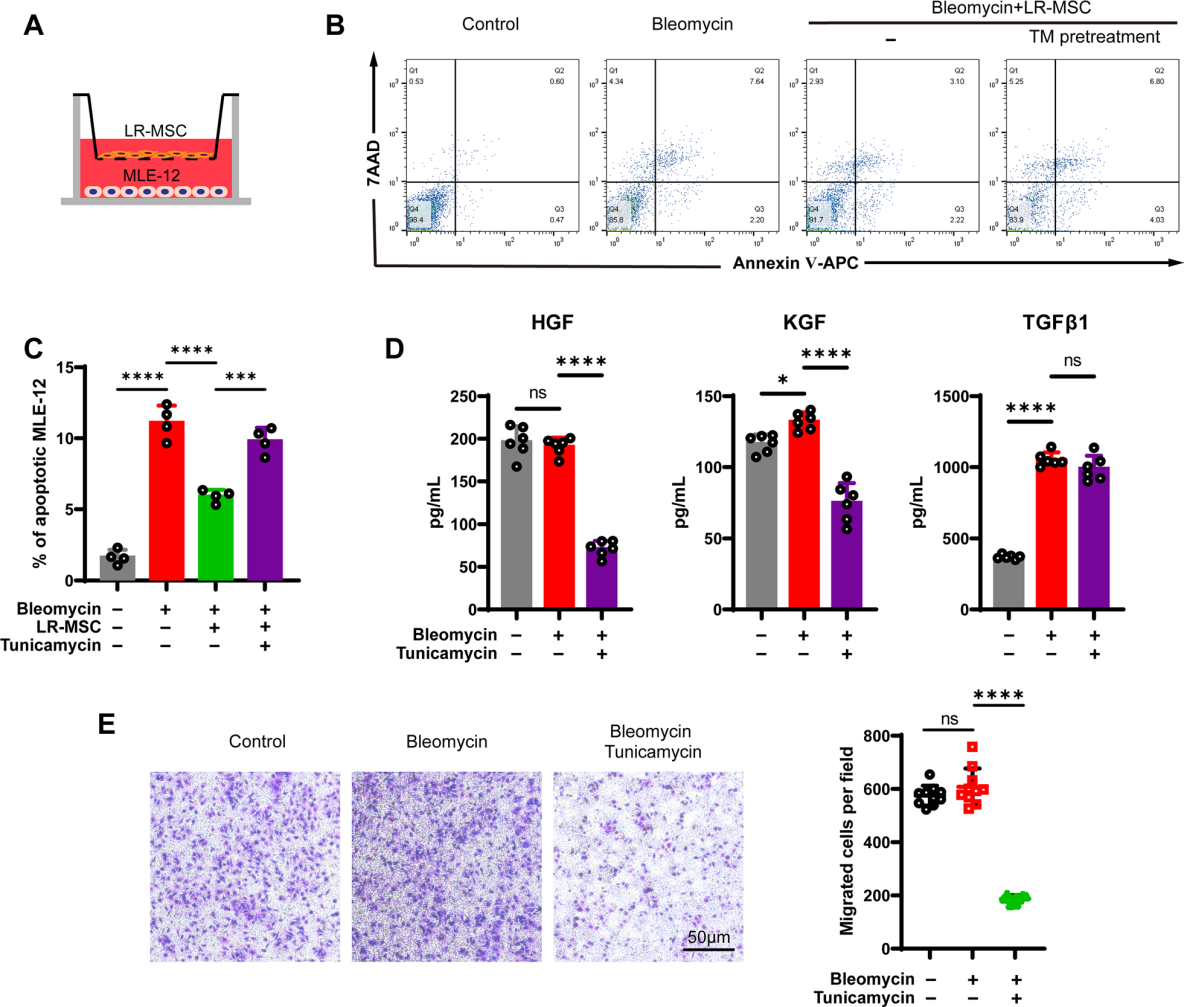
ER stress dampens the epithelium repair function of LR-MSC. **A** Schematic illustration of transwell coculture of MLE-12 and LR-MSC. **B**, **C** show the apoptosis level of cocultured MEL-12 with LR-MSC. **D** The concentration of HGF, KGF, and TGFβ1 in the coculture medium was evaluated by ELISA. **E** Transwell migration assay was performed to compare the migratory capacity of TM-pretreated LR-MSC. The left panel shows the counts of migrated LR-MSC by Giemsa staining

MSC from various origins could maintain alveolar epithelial cells (AEC) homeostasis after injuries. Using the Transwell system, we cocultured LR-MSC and mouse type 2 AEC MLE-12 in the presence of bleomycin to simulate the injury niche (Fig. 3A). The cytotoxic effects of canonical ER stress inducer tunicamycin are dose-dependent (Additional file 3: Figure E3A). To exclude the effect of apoptosis, a low concentration of tunicamycin (100 ng/mL), which did not induce significant apoptosis of LR-MSC (Additional file 3: Figure E3B), was applied for ER stress induction. LR-MSC coculture alleviated bleomycin-induced MLE-12 apoptosis, while tunicamycin preincubation of LR-MSC weakened its epithelium repair function (Fig. 3B, C). In terms of mechanism, the trophic factors in the medium, such as KGF and HGF, decreased (Fig. 3D). However, the profibrotic factor TGFβ1 did not decrease. LR-MSC achieves its epithelium repair effect by migrating to the site of injury. In the coculture condition, we found that tunicamycin pretreatment impaired the migratory ability of LR-MSC (Fig. 3E). The above results suggest that ER stress can disrupt the nutritional and repairing effects of LR-MSC on epithelial cells.

### CHOP overexpression facilitates TGFβ1-induced myofibroblast differentiation of LR-MSC

**Fig. 4:**
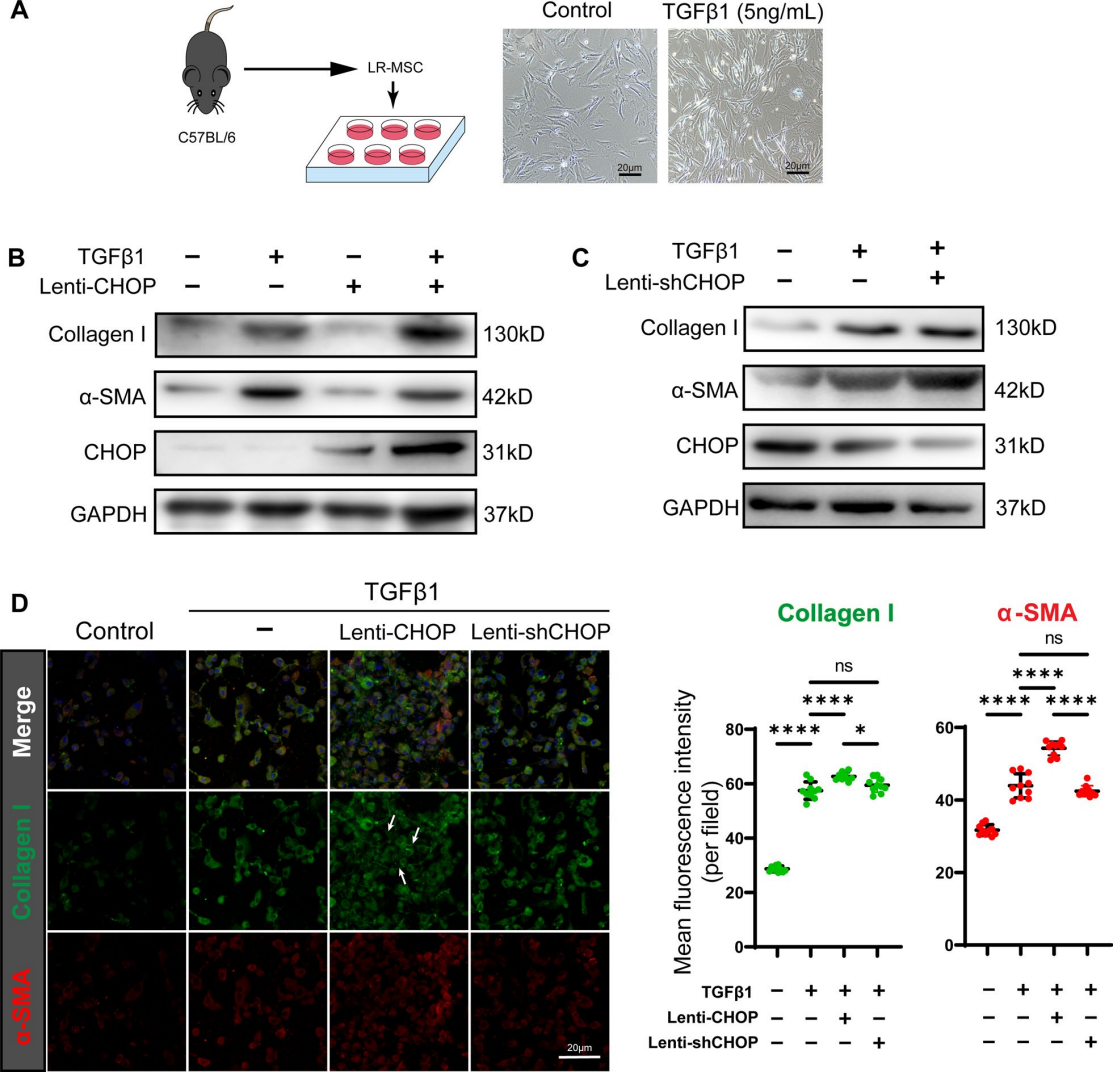
CHOP overexpression facilitates the TGFβ1-induced myofibroblast differentiation of LR-MSC. **A** Isolated LR-MSC was cultured in 6-well plates and incubated with 5 ng/mL TGFβ1 for 48 h. The shape of TGFβ1-treated LR-MSC changed and showed characters of myofibroblasts with thin and elongated appearance under phase-contrast microscopy. **B** Western blots show the effects of CHOP overexpression by Lenti-CHOP on TGFβ1-induced myofibroblast differentiation. **C** Western blots show the effects of CHOP knockdown by Lenti-shCHOP on TGFβ1-induced myofibroblast differentiation. **D** Immunofluorescence of LR-MSC for the expression of Collagen I and α-SMA under Lenti-CHOP or Lenti-shCHOP conditions. Arrows indicate the extracellular collagen fibers

To explore the relationship between ER stress and myofibroblast transformation of LR-MSC during lung fibrosis, we introduced TGFβ1, a classic profibrotic growth factor, and tunicamycin (TM) to induce myofibroblast transformation and ER stress of LR-MSC in vitro, respectively. The TGFβ1 induced a change in MSC morphology from stellate to a more thin and elongated form that resembled myofibroblasts (Fig. 4A). We found that TGFβ1 stimulation alone could not provoke significant ER stress, for the expression of Grp78 and CHOP was not increased even in the situation with a high concentration of TGFβ1 (up to 20 ng/mL) (Additional file 4: Figure E4A), indicating that myofibroblast transformation was not the reason for ER stress during pulmonary fibrosis. Tunicamycin pretreatment stimulated CHOP expression but failed to promote, even inhibit, subsequent TGFβ1-induced myofibroblast differentiation (Additional file 4: Figure E4B, C).

CHOP is a transcriptive factor associated with stem cell differentiation. To exclude the sophisticated implication between ER stress and multiple intracellular processes, we evaluated the effect of CHOP in the process of LR-MSC differentiation. CHOP overexpression by Lenti-CHOP did not promote LR-MSC apoptosis (Additional file 5: Figure E5A). Lenti-CHOP alone could not increase the expression of α-SMA and Collagen I in the absence of TGFβ1 through Western blot (Fig. 4B). However, at the presence of TGFβ1 stimulation, Lenti-CHOP strengthens its effects on myofibroblast transformation of LR-MSC as evidenced by the increased Collagen I and α-SMA (Fig. 4B) and deposition of extracellular collagen fibers (Fig. 4D). We also evaluated the effect of CHOP in basal level (physiological condition without ER stress) by CHOP knockdown of LR-MSC. Knockdown of CHOP was achieved by shRNA specifically targeting CHOP mRNA (Additional file 5: Figure E5B). We found that CHOP was dispensable for LR-MSC transformation without ER stress (Fig. 4C, D).

**Fig. 5:**
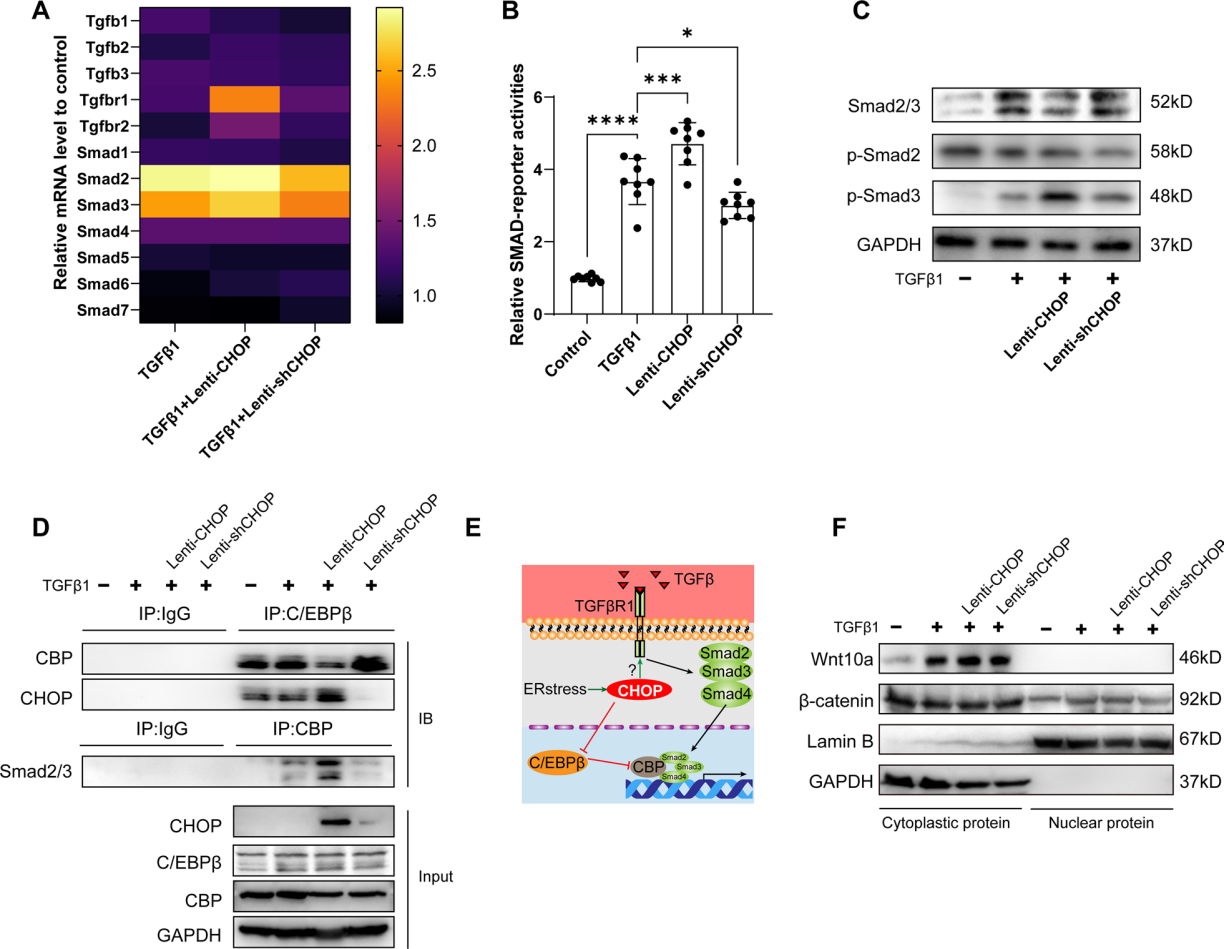
CHOP overexpression facilitates the TGFβ1-induced myofibroblast differentiation of LR-MSC by influencing the TGFβ/SMAD signaling pathway. **A** Heat map for the mRNA level of TGFβ/SMAD signaling-associated genes by qPCR. Data are shown relative to untreated LR-MSC. **B** The activation of TGFβ1/SMAD signaling was evaluated by the transfection LR-MSC with SMAD reporter plasmid using luciferase assay. **C** The level of Smad2/3 or phosphorylated Smad2/3 was evaluated by Western blot. **D** Co-IP analysis for the binding of C/EBPβ with CBP and CHOP, and binding of CBP with Smad2/3. **E** The schematic diagram illustrates the molecular mechanism by which CHOP promotes the transcriptional activity of the SMAD complex by affecting the competitive binding of C/EBPβ and Smads to CBP. **F** The effects of CHOP interference on the Wnt/β-catenin signaling pathway were evaluated by Western blot

### CHOP overexpression promotes myofibroblast differentiation of LR-MSC by sensitizing TGFβ/SMAD pathway, but not the Wnt/β-catenin signaling pathway

To elucidate the molecular mechanisms by which CHOP promotes LR-MSC transformation, we separately examined the signaling pathways associated with LR-MSC differentiation, including TGFβ/SMAD and Wnt/β-catenin signaling pathways.

qPCR analysis of key gene in the TGFβ/SMAD pathway shows increased transcription of Smad2/3 after TGFβ1 stimulation. Moreover, TGFβR1 upregulated in Lenti-CHOP-treated LR-MSC compared to Lenti-shCHOP-treated LR-MSC (Fig. 5A). The Smad2 and Smad3 transcription also increased but no difference after CHOP overexpression. Next, we found increased Smad2/3/4 activation by transducing SMAD luciferase reporter plasmid containing multiple SMAD-responsive elements in Lenti-CHOP-treated LR-MSC (Fig. 5B). This activation effect was verified by the increased phosphorated Smad3 using Western blot (Fig. 5C), which indicates the effect of CHOP overexpression on activating the TGFβ/SMAD pathway by increasing phosphorylated Smad3.

CHOP is an inhibitor of the transcription factor C/EBP family. CREBBP (CBP) acts as a co-factor assumed with the SMAD complex for downstream transcription and cooperates with C/EBPβ [23]. There is no alteration in CBP level after intervening CHOP, whereas in the Lenti-CHOP-treated LR-MSC, the binding of CHOP and C/EBPβ increased (Fig. 5D). On the contrary, the binding of CBP and Smad2/3 increased, thus promoting SMAD signaling pathway activation at the transcription level. So, C/EBPβ and Smad complex compete for CBP, while CHOP overexpression breaks the balance and favors CBP binding to Smad complex. In summary, overexpression of CHOP in LR-MSC strengthens TGFβ/SMAD pathway either by promoting Smads phosphorylation or by the co-factor binding (Fig. 5E).

Wnt/β-catenin signaling pathway was also involved in stem cell differentiation, including LR-MSC. TGFβ1 induced Wnt10a upregulation. However, the alteration in CHOP level does not influence the level of Wnt10a expression or β-catenin nuclear translocation, which is a hallmark of Wnt/β-catenin signaling pathway activation (Fig. 5F).

### CHOP knockdown improves therapeutic efficacy of LR-MSC on bleomycin-induced pulmonary fibrosis by inhibiting myofibroblast differentiation

**Fig. 6:**
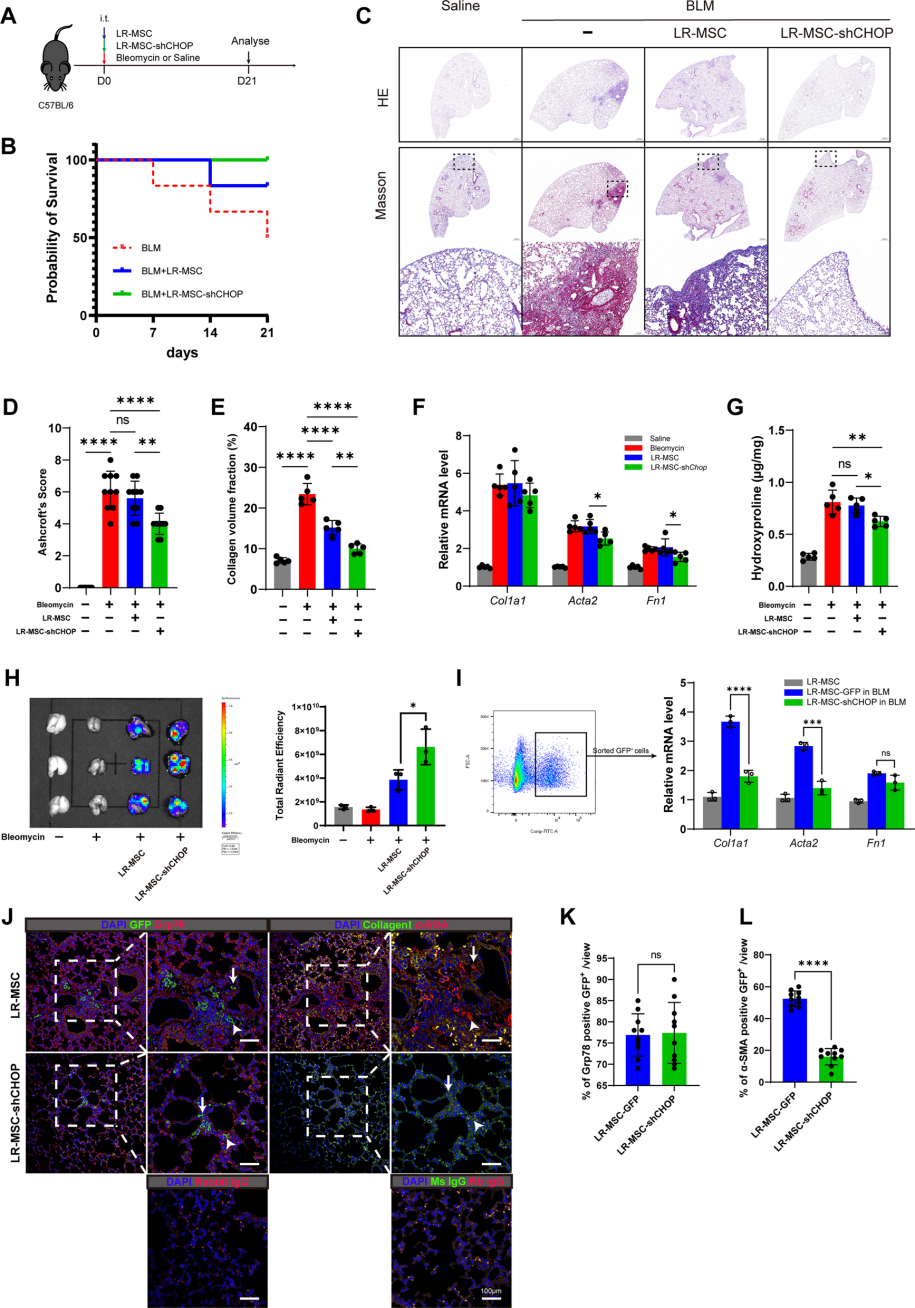
CHOP knockdown inhibits the myofibroblast differentiation of LR-MSC in vivo and moderates bleomycin-induced pulmonary fibrosis. **A** Schematic of the workflow used to treat mice. **B** Kaplan–Meier survival plot for mice treated with bleomycin (*n* = 6 per group). **C** Representative image for HE and Masson’s staining for saline, bleomycin, and LR-MSC treated mice after 21 days. **D** Ashcroft’s score (fibrosis) and **E** collagen volume fraction in different groups were shown. **F** mRNA level for fibrosis-associated genes was quantified by qPCR. **G** Hydroxyproline contents in lung tissue were quantified by ELISA. **H** Fluorescence intensity for LR-MSC-expressed GFP in different groups was tested by IVIS lumina II. **I** The mRNA level for fibrosis-associated genes in sorted GFP^+^ LR-MSC were tested by qPCR. **J** Immunofluorescence for the consecutive slides of LR-MSC and LR-MSC-shCHOP-treated BLM mouse lung showed the expression of Grp78, α-SMA, and Collagen I in GFP-positive LR-MSC. Arrows and arrowheads in different panels indicate the same LR-MSC, respectively. **K** Comparison for the counts of α-SMA-positive and **L** Grp78-positive LR-MSC in each filed

MSC has been evaluated in preliminary trials to treat pulmonary fibrosis, but the efficacy is not defined. To track the LR-MSC in the condition of pulmonary fibrosis, we established GFP-labeled LR-MSC for intratracheal instillation (i.t.) to BLM mice along with bleomycin administration (Fig. 6A). Although the number gradually decreases with time, instilled LR-MSC survived and resident in lung until 21 days post-bleomycin administration (Additional file 6: Figure E6A). LR-MSC-shCHOP improved the survival rate of BLM in 21 days (Fig. 6B). However, after receiving exogenous LR-MSC, pulmonary fibrosis did not show significant alleviation (Fig. 6C, D), besides less collagen content (Fig. 6E). Strikingly, compared to LR-MSC-GFP, the installation of LR-MSC-shCHOP alleviates pulmonary fibrosis reflected by fibrosis score of HE staining (Fig. 6D) and fibrotic area of Masson’s staining (Fig. 6E). LR-MSC-shCHOP also inhibits the ECM production compared to LR-MSC-GFP, reflected by the mRNA level of α-SMA (*Acta2*), Fibronectin (*Fn1*), and Collagen I (*Col1a1*) in the lung (Fig. 6F), as well as hydroxyproline content (Fig. 6G).

Next, we tracked the behavior of transplanted LR-MSC. Lenti-shCHOP facilitates LR-MSC survival after 21 days of bleomycin administration (Fig. 6H). The instilled LR-MSC was grafted and redistributed in the lung parenchyma, including perivascular (Additional file 6: Figure E6B), parabronchial (Additional file 6: Figure E6C), and alveolar interstitium (Fig. 6J). These exogenous LR-MSC show aggregated properties, suggesting in situ proliferation (Additional file 6: Figure E6D). We also examined the mRNA level of *Acta2* and *Col1a1* in sorted GFP^+^ LR-MSC. Results showed a lower *Acta2*, *Fn1*, and *Col1a1* in LR-MSC-shCHOP than LR-MSC-GFP in BLM mice (Fig. 6I), which suggested less myofibroblast differentiation. Immunofluorescence of lung tissue also showed extensive colocalization of GFP with Grp78, α-SMA, and Collagen I (Fig. 6J), indicating that these supplied exogenous LR-MSC suffered from ER stress and transformed to myofibroblast, which is the likely cause of the failure of treatment for pulmonary fibrosis. While in LR-MSC-shCHOP treated BLM mouse, the LR-MSC-shCHOP also suffered from ER stress with no difference in the level for Grp78 (Fig. 6K), the α-SMA-positive LR-MSC decreased compared to LR-MSC-GFP (Fig. 6L).

## Discussion

**Fig. 7:**
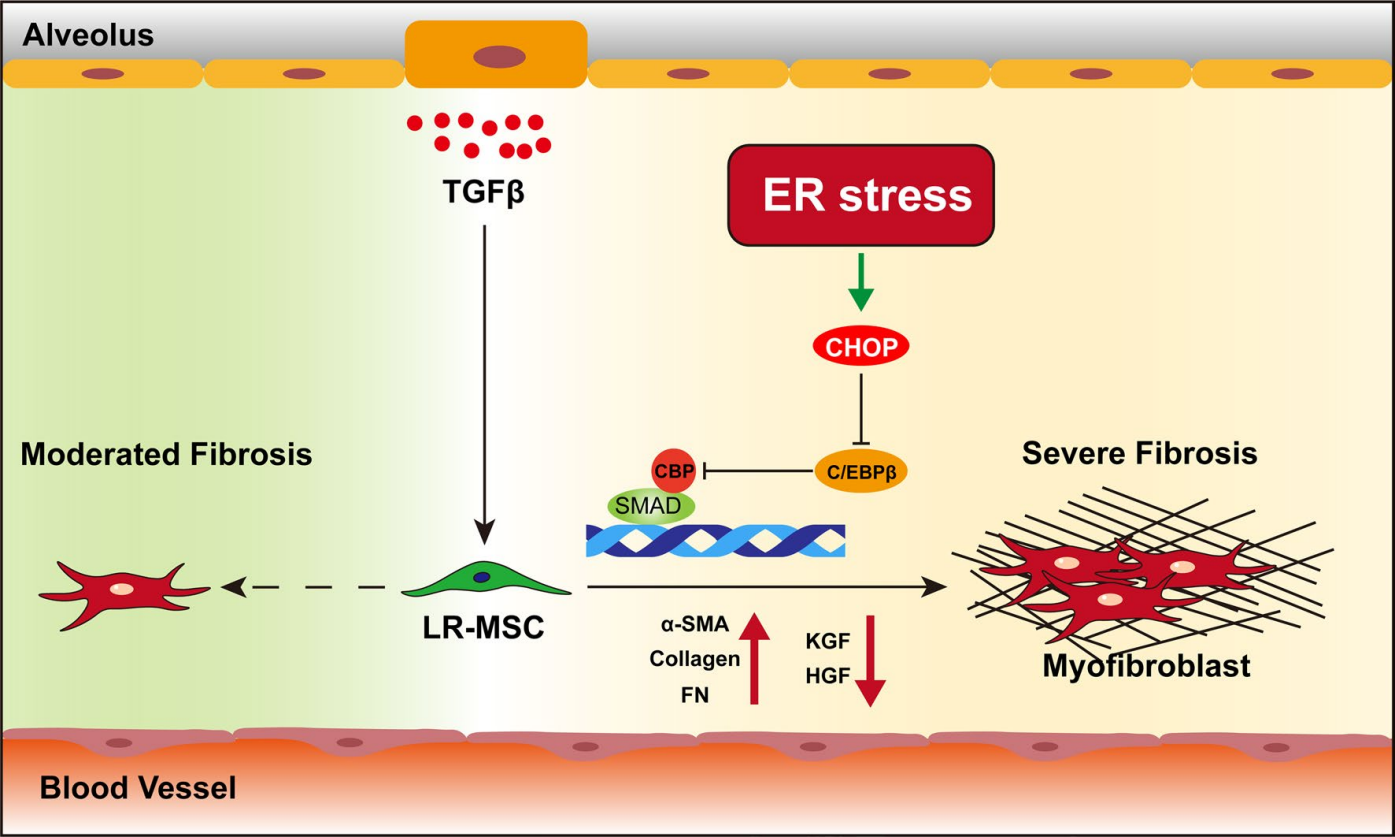
Schematic illustration of the mechanism by which ER stress is involved in pulmonary fibrosis by modulating myofibroblast differentiation of LR-MSC. On the one hand, ER stress during pulmonary fibrosis dampens the alveolar repair function of LR-MSC; on the other hand, it facilitates the activation of the TGFβ/SMAD signaling pathway and promotes the myofibroblast differentiation of LR-MSC to produce more ECM.

Our work demonstrated that LR-MSC transformed to myofibroblasts and was involved in IPF pathogenesis. Meanwhile, this transition was accompanied by ER stress, which impaired the normal epithelium repair function of LR-MSC. Importantly, we found that the ER stress downstream transcription factor CHOP modifies LR-MSC transformation (Fig. 7). Overexpression of CHOP facilitates the TGFβ1-induced myofibroblasts differentiation of LR-MSC in vitro. On the contrary, downregulation of CHOP protects mice from BLM-induced pulmonary fibrosis by preventing LR-MSC transformation in vivo.

Despite various pathogenic factors, pulmonary fibrosis is a common outcome resulting from an abnormal repair process characterized by excessive proliferation and activation of mesenchymal cells following epithelial or endothelial cell injury. Inhibition of stromal hyperplasia is one of the strategies for treating pulmonary fibrosis. Although this inhibition could not rescue or regenerate the lost epithelial cells, neglect of it will lead to the remodeling of normal lung tissue and irreversible lung destruction. The mechanisms underlying the activation and proliferation of mesenchymal cells are not yet fully understood, thus limiting the development of anti-fibrosis medicine and therapies, and thus pulmonary fibrotic diseases, such as IPF, remain irreversible and incurable.

Myofibroblasts are populations that combine ECM production with contractile properties similar to those of myocytes, and it was considered the most active cell type that participates in pulmonary fibrosis. The conventional view about the source of myofibroblasts during pulmonary fibrosis is primarily fibroblasts colonized in the lung, and subsequent studies have found that pericyte-derived myofibroblasts outnumber those of fibroblast [24]. In the recent decade, MSC was found to colonize in the stromal of various postnatal organs, including the lung (LR-MSC), and also acts as precursor cells to provide fibroblasts or myofibroblast during fibrosis, thus attracting people’s attention [25, 26]. Consistent with these findings, in our work, we found LR-MSC-derived myofibroblasts aggregated in fibroblastic foci with typical morphological presentation both in IPF patients (Fig. 1B) and in BLM model (Fig. 2A). The aggregated distribution characteristic suggests that LR-MSC undergoes proliferation. Previous studies demonstrated that LR-MSC colonized perivascularly [20, 26–28]. Our results showed that in IPF lung, besides fibroblastic foci, the CD90^+^ stromal cells aggregated in perivascular areas (Fig. 1C) and, in the BLM model, distribute peribronchiolar (Fig. 2A). It is conceivable that LR-MSC migrates to the injured alveolar cells and proliferates, differentiates during pulmonary fibrosis, for LR-MSCs are sparse and dispersed in the physiological state and thus difficult to observe in the alveolar septum.

To further study the biological behavior and undermined mechanisms for this pathogenic transition of LR-MSC, reliable primary cell identification and isolation methods are necessary for in vitro culture. Methods to isolate human LR-MSC mainly include cloned expansion of BALF-derived or lung tissue outgrowth cells adherent to plastic dishes [29]. For murine LR-MSC isolation, the cellular fraction of Sca1^+^CD45^−^CD31^−^ from lung digest was a widely accepted panel [22, 30–32]. However, as a pan-stem cell surface marker, Sca1 could not distinguish progenitor cells for different lineage. For example, the recently identified bronchioalveolar stem cells (BASC) have the same phenotype for Sca1^+^CD45^−^CD31^−^ but can only regenerate descendants of epithelial lineages, such as club cells, ciliated cells, and alveolar epithelial cells [33, 34]. We found that approximately 8% of the Sca1^+^CD45^−^CD31^−^ cell population expressed epithelial markers EpCam (Fig. 2C), consisting of the phenotype of epithelial stem cells including BASC. The heterogeneity in components of isolated LR-MSC may explain the ex vivo alveolar epithelial cells differentiation of LR-MSC in some studies [21, 35, 36].

In situ immunofluorescence or ex vivo Western blot for isolated primary LR-MSC confirmed the transformation and ER stress of LR-MSC mirrored to IPF in bleomycin-induced mice pulmonary fibrosis. In BLM mice, we also noticed a decrease in the number of LR-MSC, consistent with the finding by Jun et al*.* [37], but the explanation for this is uncertain. One possible cause is apoptosis of LR-MSC, as we also detected an upregulation of the apoptogenic transcription factor CHOP. Another reason could be the loss of Sca1 expression during the transformation in MSCs, resulting in a reduction in the number of MSCs we isolate by immunophenotyping.

To reveal the effect of ER stress on the repair function of LR-MSC, we cocultured tunicamycin-induced LR-MSC with alveolar epithelial cells. Our results suggest that ER stress can disrupt the repair function of LR-MSC in three aspects: growth factor secretion, epithelial cell resistance to apoptosis, and migration capacity (Fig. 3). Multiple external factors can cause LR-MSC to suffer from endoplasmic reticulum stress and cause impaired LR-MSC repair capacity. For example, factors that lead to ER stress like smoking [38], agricultural organic dust [39], and hyperoxia [40] can cause abnormal LR-MSC repair in humans.

In addition to losing its repair function, LR-MSC can transform into myofibroblasts, a potent profibrotic cell type. To investigate whether ER stress affects this transformation process, we conducted experiments in vitro using primary LR-MSC. Research, as well as our work, demonstrated that TGFβ1 could stimulate LR-MSC to undergo phenotype conversion to myofibroblast [41]; therefore, we used TGFβ1 to build a model of the myofibroblast transformation of LR-MSC (Fig. 4A). Although ER stress and transformation of LR-MSC exist simultaneously, it is necessary to clarify whether there is a causal relationship between the two processes. Induction of transformation by TGFβ1 did not increase Grp78 and CHOP expression (Additional file 4: Figure E4A). Therefore, the process of transformation does not induce ER stress. On the contrary, the addition of tunicamycin did not show increased myofibroblast differentiation reflected by the expression of α-SMA and Collagen I (Additional file 4: Figure E4B, C). Given the critical role of CHOP in multiple biological processes, we modulated the expression of CHOP (by overexpression or knockdown with shRNA) instead of the administration of tunicamycin, which seems to incite various biological effects other than the upregulation of CHOP. Although upregulation of CHOP during unrelieved ER stress promotes apoptosis, overexpression of CHOP alone does not cause significant apoptosis (Additional file 5: Figure E5A), which was inconsistent in alveolar epithelial cells [42]*.* We found that the overexpression of CHOP facilitates the TGFβ1-induced myofibroblast differentiation (Fig. 4B, D). Knockdown of CHOP does not influence transformation (Fig. 4C, D) because CHOP keeps at a low level without ER stress.

We further sought to elucidate the molecular mechanisms by which CHOP promotes transformation. Firstly, we examined the changes in the expression of TGFβ/SMAD signaling pathway-related components after interfering with CHOP expression by qPCR. We found that the components that changed significantly after the intervention was mainly Smad2 and Smad3 and elevated TGFβR2 after overexpression of CHOP (Fig. 5A). Conditional knockout of TGFβR2 in lung fibroblasts has shown to attenuate BLM pulmonary fibrosis [43]. In terms of activity, overexpression of CHOP activated the SMAD signaling pathway as well as elevated phosphorylation of Smad3 (Fig. 5B, C). CHOP is a member of the CCAAT/enhancer-binding protein (C/EBP) family of transcript factors and can inhibit the function of C/EBPβ by forming a heterodimer with C/EBPβ. Evidence demonstrated that the transcriptive activity of both C/EBPβ and Smad2/3/4 was modulated by transcriptional coactivator CREB-binding protein (CBP) [23]. Our study found that in the condition of CHOP overexpression, the overall level of CBP was not influenced, and the binding between CBP and C/EBPβ decreased, while CBP and Smad2/3 increased, thus activating the SMAD signaling pathway. Therefore, we speculate that C/EBPβ and Smad2/3 would compete to bind CBP. Overexpression of CHOP would predispose CBP to bind Smads by inhibiting C/EBPβ (Fig. 5E). In addition, the Wnt/β-catenin signaling pathway involved in the myofibroblast differentiation [41, 44, 45], and the ligand Wnt10a were most upregulated prominently and activated Wnt/β-catenin signaling pathway in an auto/paracrine manner. Our study did not detect changes in Wnt/β-catenin signaling after CHOP interfering (Fig. 5F), indicating that ER stress or CHOP overexpression influenced the fate of LR-MSC independent of Wnt/β-catenin signaling.

As the preliminary use of MSC in IPF treatment, LR-MSC also attracted people’s attention to pulmonary fibrosis treatment [46]. Here, we applied the LR-MSC to bleomycin-treated mice to test whether exogenous LR-MSC alleviates pulmonary fibrosis. The transplanted LR-MSC grafted and resided in the lung but did not alleviate the pulmonary fibrosis; as similar to endogenous LR-MSC, they transformed to myofibroblast. However, when CHOP in exogenous LR-MSC was knocked down, the grafted LR-MSC increased, transformation reduced, and pulmonary fibrosis was alleviated. However, we could not exclude the influence on the immunomodulatory effect of LR-MSC. Previous studies have shown that LR-MSC suppressed T cells activity via PGE_2_ [47], regulated the balance of Th17 and Treg cells [48], and regulated the cytokines production [22]. Given that LR-MSC substantially reduced during bleomycin-induced pulmonary fibrosis [37], there is no doubt that the cellular component of immunosuppression is reduced in the lungs. Since we transplanted LR-MSC in parallel with BLM perfusion, LR-MSC must be involved in the inflammatory damage phase of fibrosis.

Clinically, IPF is more common in older men. In this study, we used male mice to establish a pulmonary fibrosis model, representing the clinical characteristics of most patients with IPF. However, women are also affected in some other causes of pulmonary fibrosis, such as lung involvement caused by certain connective tissue diseases. It would be interesting if the effect of gender on LR-MSC could be clarified by extending this study to a comparative study of LR-MSC between male and female mice.

## Conclusion

In conclusion, our and previous works have shown that LR-MSC is involved in pulmonary fibrosis. Abnormalities in LR-MSC function due to multiple factors that diminish the epithelial repair effect of MSC, such as smoking [38] and agricultural organic dust [39], might involve in the pulmonary fibrosis process through enhanced profibrotic effects of LR-MSC. In future drug development, therapies that target and modify LR-MSC function, such as monoclonal antibody-guided vector for siRNA [49], may be the strategy for treating pulmonary fibrosis by knockdown CHOP of LR-MSC. Exogenous gene-edited LR-MSC transplanted as an alternative strategy by enhancing the beneficial therapeutic effects of MSC and removing the detrimental ones, such as the expression of CHOP in our study.

## Supplementary Information

**Additional file 1**: **Figure E1**. IPF lung shows a higher level of ECM production and UPR. Western blot shows the expression of ECM production-associated protein (Collagen I and α-SMA), and UPR-associated protein including (ATF6, IRE1, PERK, Grp78, and CHOP) in non-fibrotic (normal) lung samples (n = 6) and IPF lung samples (n = 6). Relative quantification of protein expression was shown in histogram (right panel) with mean ± SEM. # represent significant difference on mean by t test (p < 0.05).

**Additional file 2**: **Figure E2**. Identification of the MSC characteristics of LR-MSC. (A) Expression of surface markers for MSC, including CD90 and CD105, and absence of hematopoietic marker CD45, CD34, and endothelial marker CD31 of LR-MSC was evaluated by FACS. A gray area represents isotype control. (B) Represented images for the adipogenic, osteogenic, and chondrogenic differentiation of LR-MSC by oil red O, alizarin red, and alcian blue staining.

**Additional file 3**: **Figure E3**. Cytotoxic effect of tunicamycin is dose-dependent. (A) Treatment LR-MSC with tunicamycin show dose-dependent cytotoxicity when concentration is over 500 ng/mL by MTT assay. (B) LR-MSC treated with 100 ng/mL of tunicamycin did not show significant apoptosis.

**Additional file 4**: **Figure E4**. Tunicamycin-induced ER stress failed to facilitate the TGFβ1-induced myofibroblast differentiation. (A) Western blot demonstrated that TGFβ1 treatment did not lead to UPR, as the chaperone Grp78 and CHOP did not upregulate compared to the positive control (TM). (B) Western blot shows the addition of tunicamycin failed to promote the expression of Collagen I and α-SMA. (C) Immunofluorescence of LR-MSC shows the addition of tunicamycin failed even inhibited the expression of α-SMA.

**Additional file 5**: **Figure E5**. The effects of overexpression or knockdown of CHOP in LR-MSC. (A) Overexpression of CHOP did not induce apoptosis of LR-MSC. (B) Western blot shows the efficiency of knockdown of CHOP by transfection LR-MSC with lentivirus-loaded nonspecific (NC) or CHOP shRNA.

**Additional file 6**: **Figure E6**. Transplanted LR-MSC i.t. survived and proliferated in the mouse lung. (A) The detection of fluorescence of GFP indicated the graft of LR-MSC in mouse lung. Furthermore, LR-MSC decreased along with time within 21 days after installation. (B) and (C) demonstrated the transplanted LR-MSC located in perivascular or parabronchial areas in the lung, respectively. The area framed by the dotted line is the blood vessel in the high magnification image and bronchus in (C). V, vessel. B, bronchus. (D) The MSCs clustered together, a feature that suggested that they proliferated in situ.

**Additional file 7**: **Table E1**. Characteristics of donors for lung tissue samples.

**Additional file 8**: **Table E2**. Mouse primer sequences for qPCR.

## Data Availability

Original data can be requested from the corresponding author.

## Abbreviations

IPF: Idiopathic pulmonary fibrosis
ECM: Extracellular matrix
LR-MSC: Lung resident mesenchymal stem cell
ER: Endoplasmic reticulum
CHOP: C/EBP homologous protein
UPR: Unfolded protein response
α-SMA: Alpha smooth muscle actin
ATF6: Activating transcription factor 6
IRE1: Inositol-requiring enzyme 1
PERK: PRKR-like endoplasmic reticulum kinase
Grp78: Glucose-regulated protein, 78 kDa
Sca1: Stem cell antigen 1
AEC: Alveolar epithelial cell
BLM: Bleomycin
TM: Tunicamycin
TGFβ: Transforming growth factor beta
CBP: CREB-binding protein
