# Supplementary figures and images for: Endoplasmic reticulum stress modulates the fate of lung resident mesenchymal stem cell to myofibroblast via C/EBP homologous protein during pulmonary fibrosis
Source: Stem Cell Res Ther. 2022 Jun 28;13:279. doi: 10.1186/s13287-022-02966-1 (PMC9241222; doi:10.1186/s13287-022-02966-1)

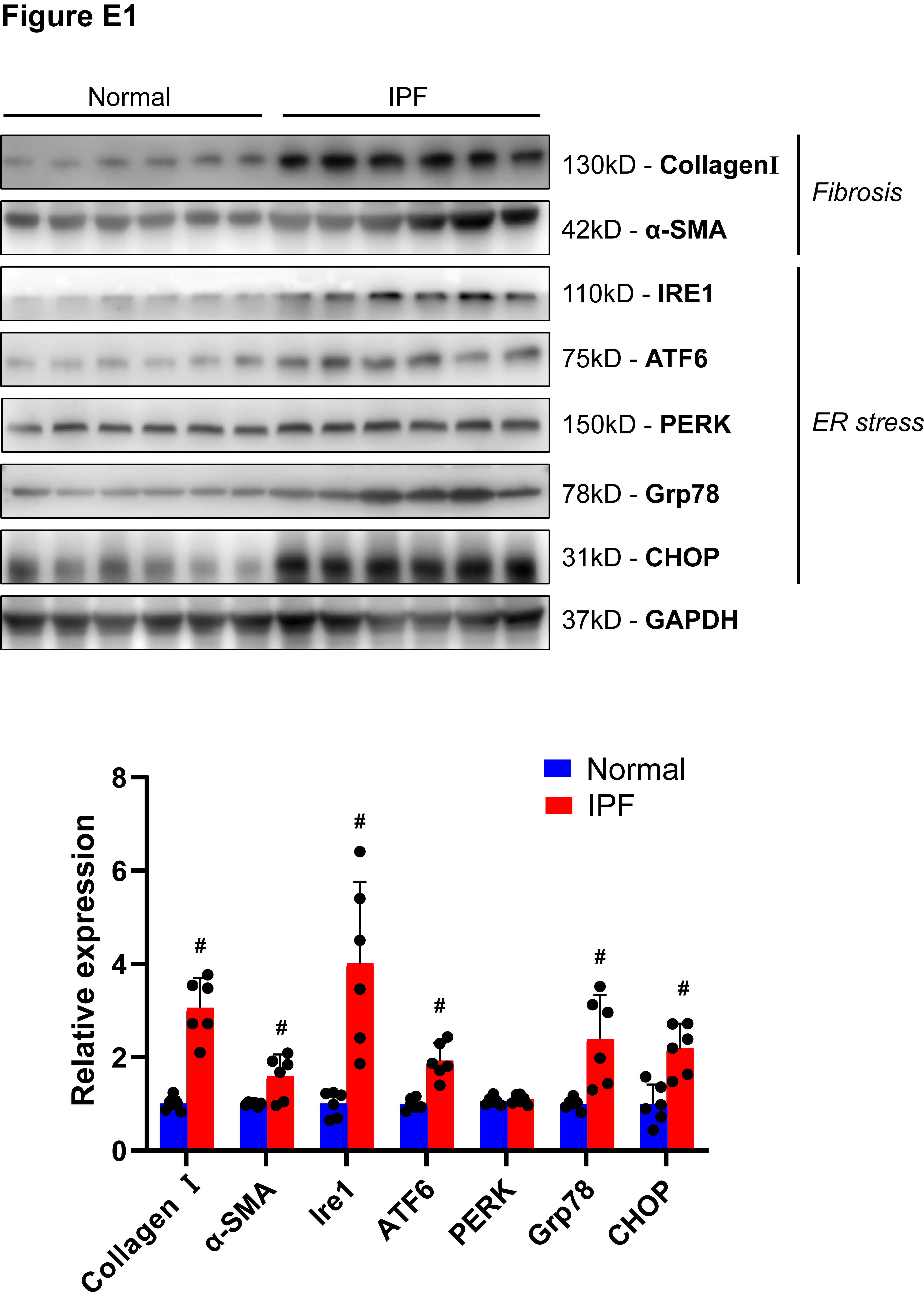

Supplement: Supplementary file 1 — Additional file 1: Figure E1. IPF lung shows a higher level of ECM production and UPR. Western blot shows the expression of ECM production-associated protein (Collagen I and α-SMA), and UPR-associated protein including (ATF6, IRE1, PERK, Grp78, and CHOP) in non-fibrotic (normal) lung samples (n = 6) and IPF lung samples (n = 6). Relative quantification of protein expression was shown in histogram (right panel) with mean ± SEM. # represent significant difference on mean by t test (p < 0.05). [file 13287_2022_2966_MOESM1_ESM.tif]

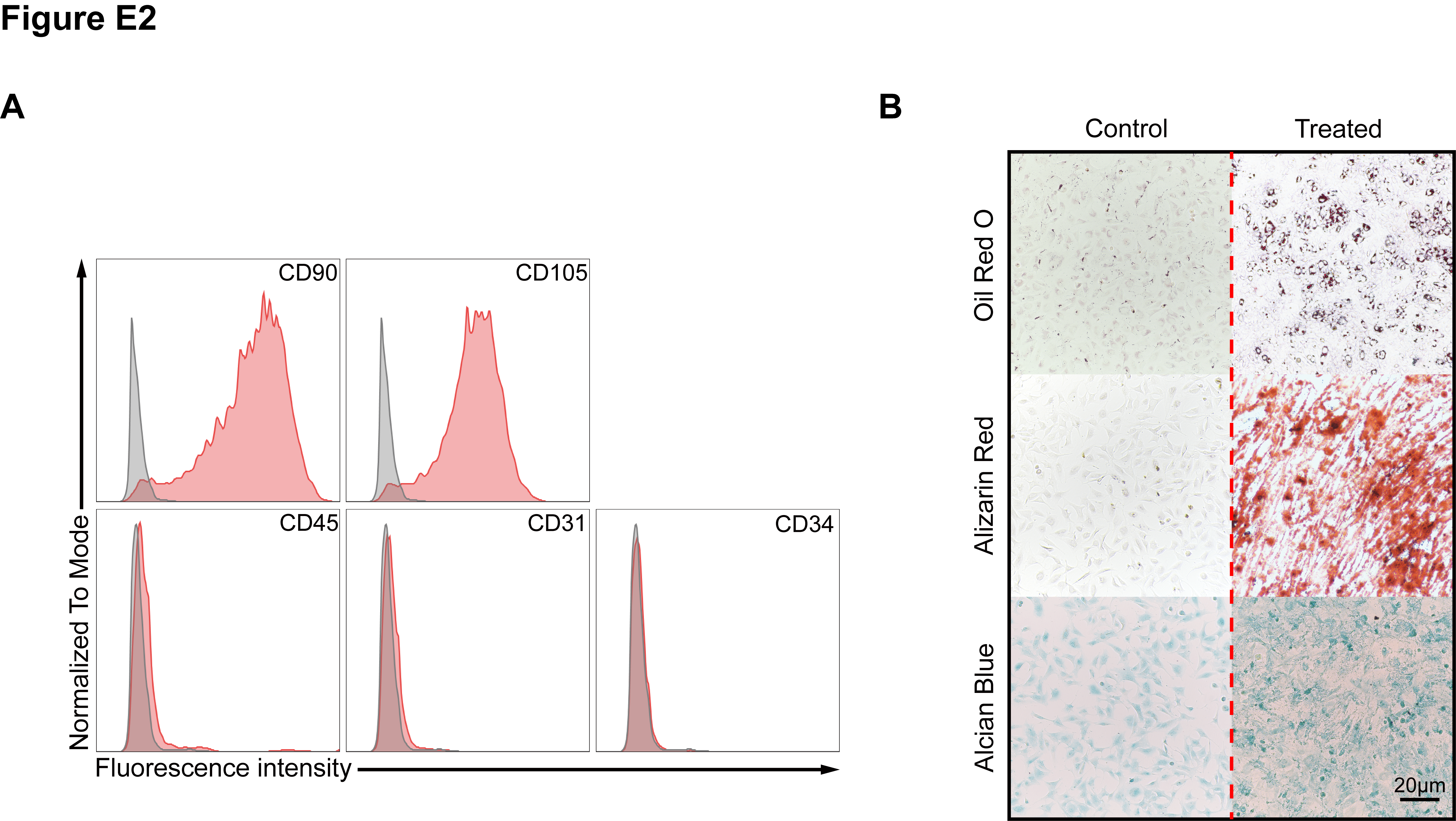

Supplement: Supplementary file 2 — Additional file 2: Figure E2. Identification of the MSC characteristics of LR-MSC. (A) Expression of surface markers for MSC, including CD90 and CD105, and absence of hematopoietic marker CD45, CD34, and endothelial marker CD31 of LR-MSC was evaluated by FACS. A gray area represents isotype control. (B) Represented images for the adipogenic, osteogenic, and chondrogenic differentiation of LR-MSC by oil red O, alizarin red, and alcian blue staining. [file 13287_2022_2966_MOESM2_ESM.tif]

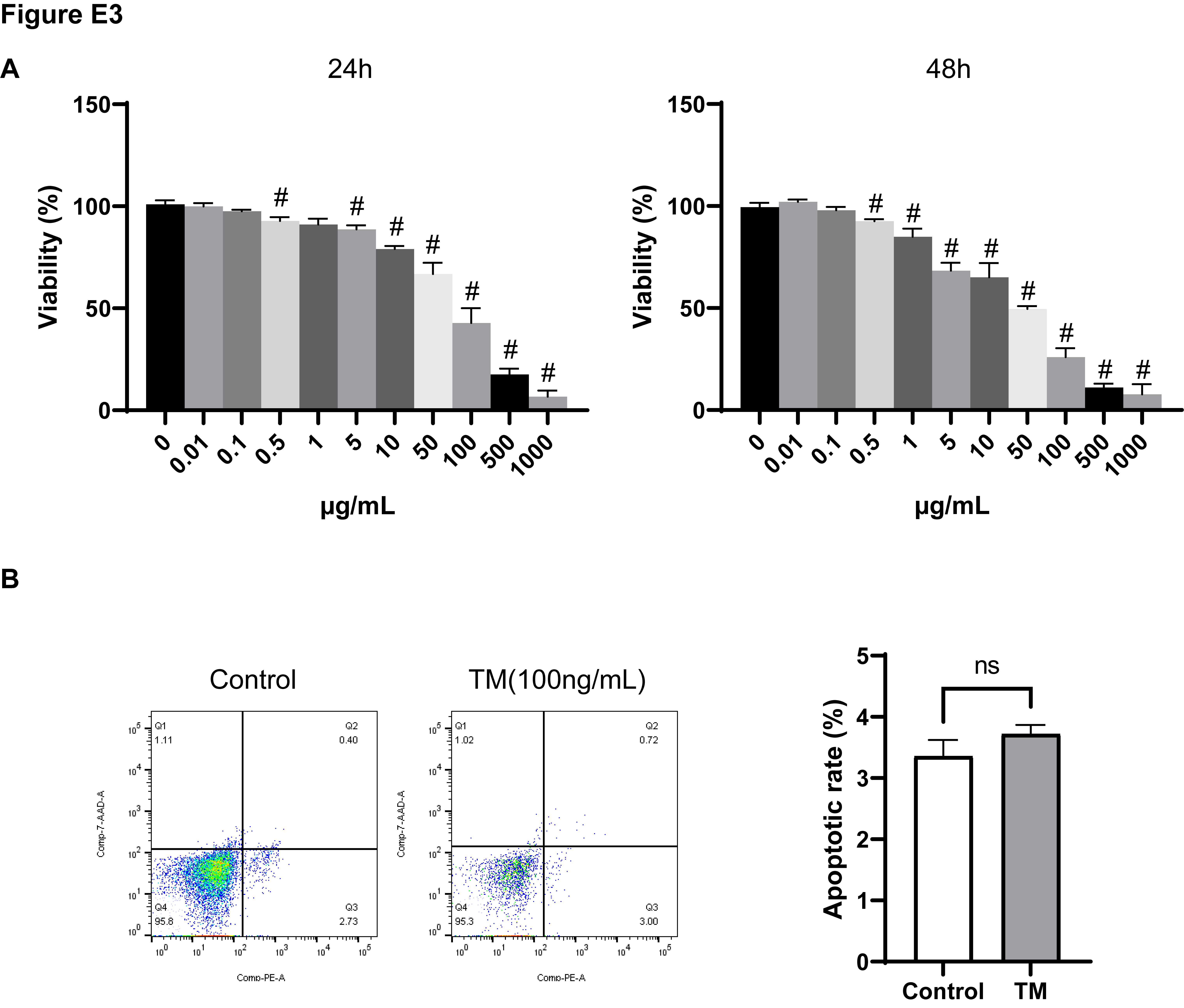

Supplement: Supplementary file 3 — Additional file 3: Figure E3. Cytotoxic effect of tunicamycin is dose-dependent. (A) Treatment LR-MSC with tunicamycin show dose-dependent cytotoxicity when concentration is over 500 ng/mL by MTT assay. (B) LR-MSC treated with 100 ng/mL of tunicamycin did not show significant apoptosis. [file 13287_2022_2966_MOESM3_ESM.tif]

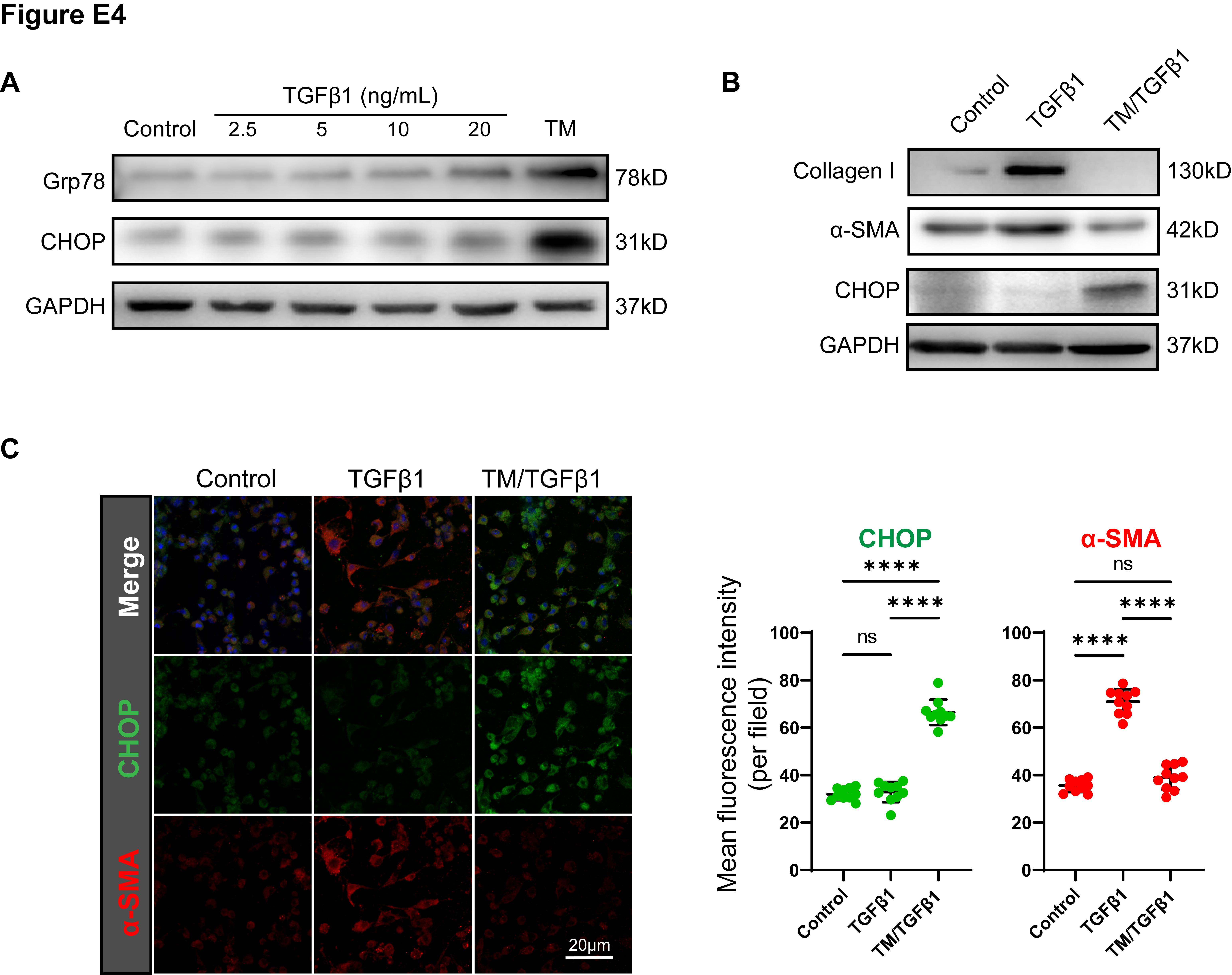

Supplement: Supplementary file 4 — Additional file 4: Figure E4. Tunicamycin-induced ER stress failed to facilitate the TGFβ1-induced myofibroblast differentiation. (A) Western blot demonstrated that TGFβ1 treatment did not lead to UPR, as the chaperone Grp78 and CHOP did not upregulate compared to the positive control (TM). (B) Western blot shows the addition of tunicamycin failed to promote the expression of Collagen I and α-SMA. (C) Immunofluorescence of LR-MSC shows the addition of tunicamycin failed even inhibited the expression of α-SMA. [file 13287_2022_2966_MOESM4_ESM.tif]

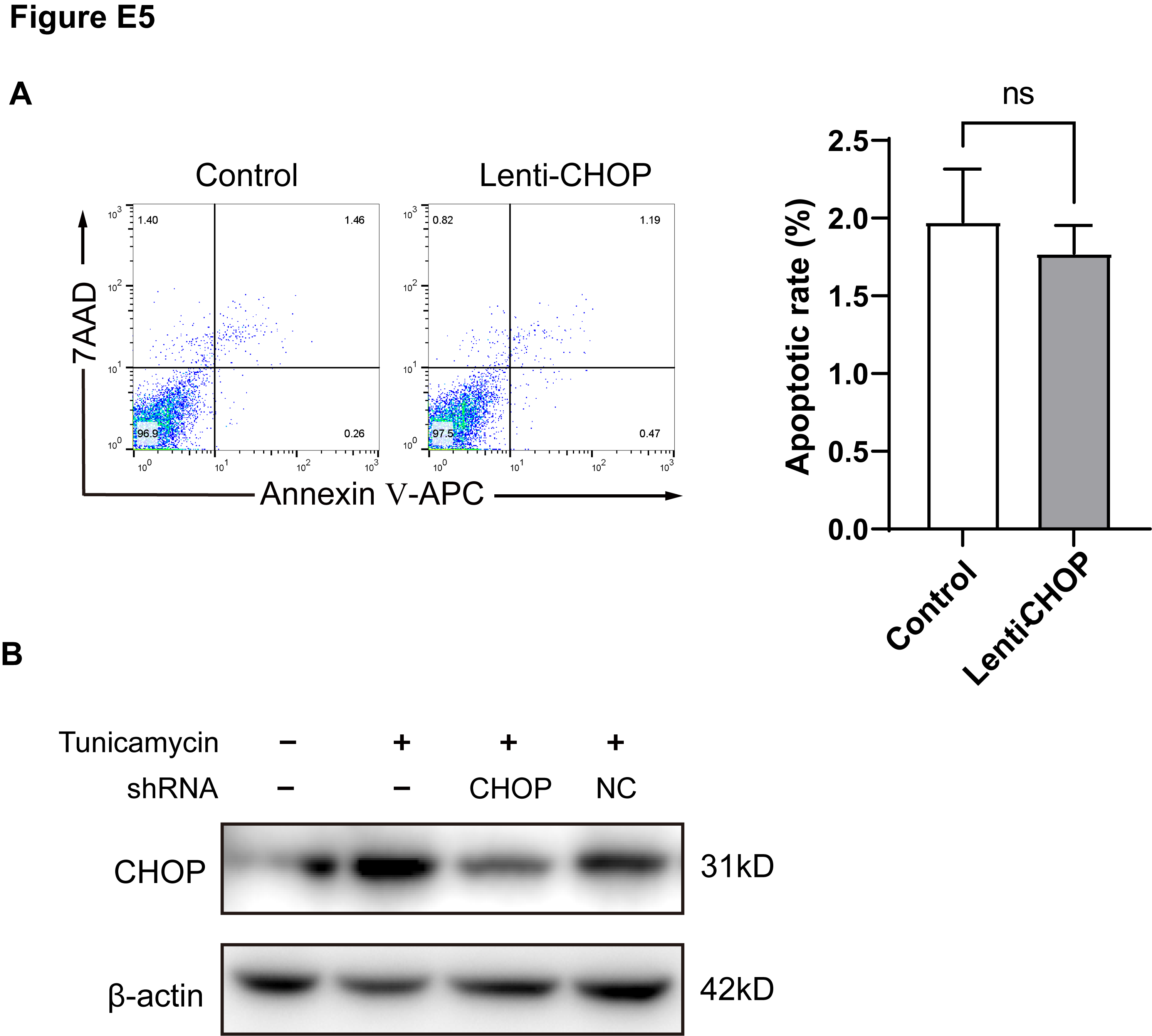

Supplement: Supplementary file 5 — Additional file 5: Figure E5. The effects of overexpression or knockdown of CHOP in LR-MSC. (A) Overexpression of CHOP did not induce apoptosis of LR-MSC. (B) Western blot shows the efficiency of knockdown of CHOP by transfection LR-MSC with lentivirus-loaded nonspecific (NC) or CHOP shRNA. [file 13287_2022_2966_MOESM5_ESM.tif]

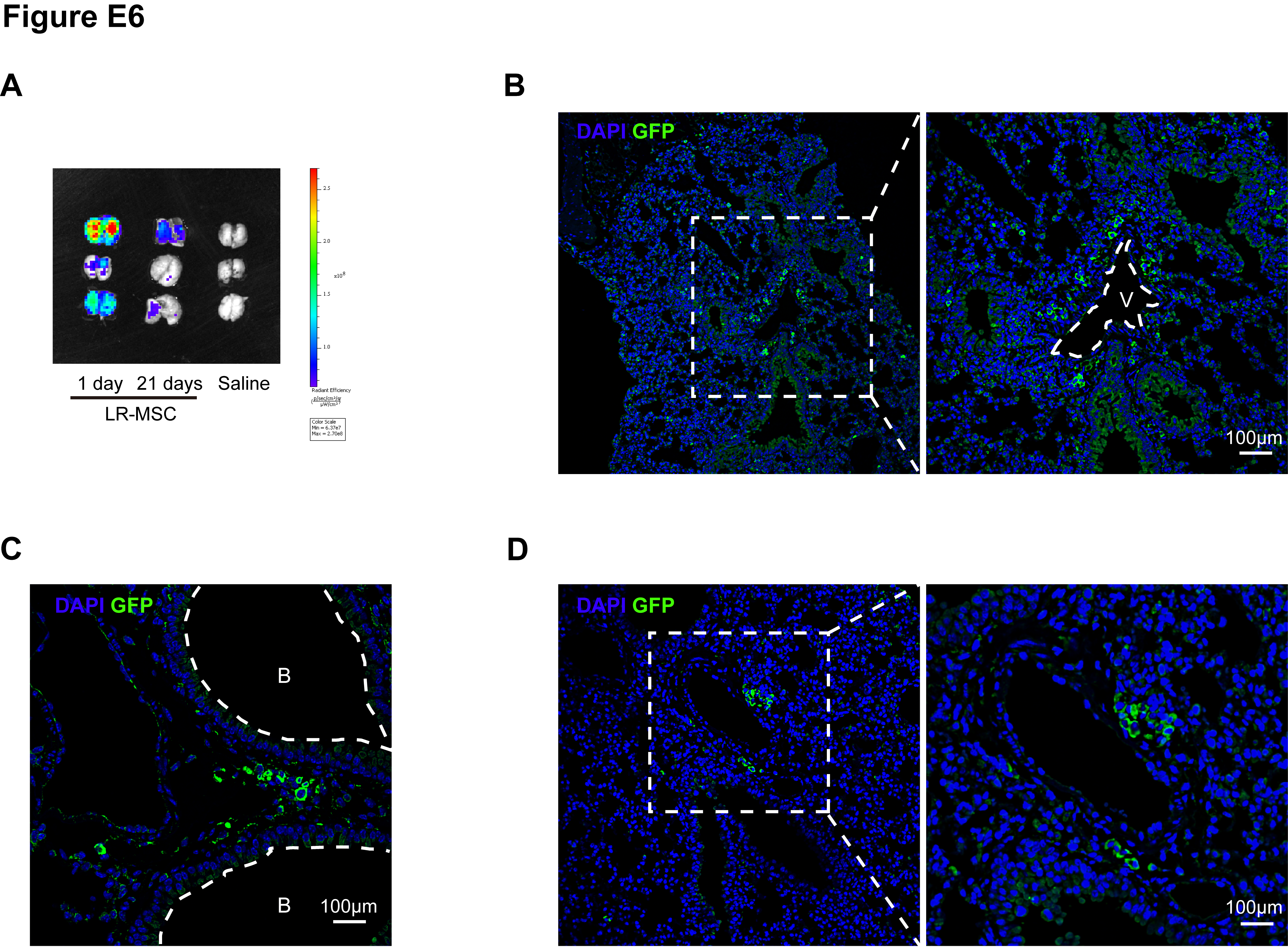

Supplement: Supplementary file 6 — Additional file 6: Figure E6. Transplanted LR-MSC i.t. survived and proliferated in the mouse lung. (A) The detection of fluorescence of GFP indicated the graft of LR-MSC in mouse lung. Furthermore, LR-MSC decreased along with time within 21 days after installation. (B) and (C) demonstrated the transplanted LR-MSC located in perivascular or parabronchial areas in the lung, respectively. The area framed by the dotted line is the blood vessel in the high magnification image and bronchus in (C). V, vessel. B, bronchus. (D) The MSCs clustered together, a feature that suggested that they proliferated in situ. [file 13287_2022_2966_MOESM6_ESM.tif]
