# Supplementary material for: Endoplasmic reticulum stress modulates the fate of lung resident mesenchymal stem cell to myofibroblast via C/EBP homologous protein during pulmonary fibrosis
Source: Stem Cell Res Ther. 2022 Jun 28;13:279. doi: 10.1186/s13287-022-02966-1 (PMC9241222; doi:10.1186/s13287-022-02966-1)
Supplement: Supplementary file 7 — Additional file 7: Table E1. Characteristics of donors for lung tissue samples. [file 13287_2022_2966_MOESM7_ESM.docx]

**Supplemental Table E1**. Characteristics of donors for lung tissue samples

|  | Normal (n=6) | IPF (n=6) | *P* |
| --- | --- | --- | --- |
| Gender (M/F) | 4/2 | 6/0 | 0.121^*^ |
| Age (year) | 65.3±13.7 | 73.7±6.5 | 0.205^†^ |
| Body mass index (kg/m^2^) | 26.62±6.8 | 24.26±10.5 | 0.657^†^ |
| Smoker (n) | 4 | 3 | 0.558^*^ |
| Pack-years of smoker | 54.25±9.251 | 49.33±4.041 | 0.436^†^ |
| FEV1, % ref | 93.72±4.653 | 85.07±5.286 | 0.013^†^ |
| FEV1/FVC, % | 91.82±3.604 | 85.11±5.341 | 0.029^†^ |
| DLco, % ref | 92.13±2.021 | 36.70±5.874 | <0.0001^†^ |
| Clinical history (%) |  |  |  |
| NSCLC | 83.3 | 0 | 0.0034^*^ |
| SCLC | 16.7 | 0 | 0.2963^*^ |
| Radiological findings (%) |  |  |  |
| Honeycomb | 0 | 100 | 0.0005^*^ |
| Reticular opacities | 0 | 100 | 0.0005^*^ |
| Traction bronchiectasis | 0 | 100 | 0.0005^*^ |
| Ground glass | 50.0 | 16.7 | 0.2207^*^ |
| Consolidation | 83.3 | 33.3 | 0.0790^*^ |

*. The chi-square test was used to compare the differences in composition ratio; †. Values are shown as mean ± SD. Comparison of mean value between two groups using two-tailed *t*-test. The statistically significant difference when *p*＜0.05. FEV1, forced expiratory volume in 1 second; FVC, forced vital capacity; DLco, carbon monoxide diffusion capacity; NSCLC, non-small cell lung cancer.
