# Supplementary material for: Endoplasmic reticulum stress modulates the fate of lung resident mesenchymal stem cell to myofibroblast via C/EBP homologous protein during pulmonary fibrosis
Source: Stem Cell Res Ther. 2022 Jun 28;13:279. doi: 10.1186/s13287-022-02966-1 (PMC9241222; doi:10.1186/s13287-022-02966-1)
Supplement: Supplementary file 8 — Additional file 8: Table E2. Mouse primer sequences for qPCR. [file 13287_2022_2966_MOESM8_ESM.docx]

**Supplementary Table E2**. Mouse Primer for qPCR

| Target Gene | Sequence (5’-3’) |
| --- | --- |
| *Col1a1* | Forward: GCTCCTCTTAGGGGCCACT |
|  | Reverse: CCACGTCTCACCATTGGGG |
| *Acta2* | Forward: GTCCCAGACATCAGGGAGTAA |
|  | Reverse: TCGGATACTTCAGCGTCAGGA |
| *Fn1* | Forward: ATGTGGACCCCTCCTGATAGT |
|  | Reverse: GCCCAGTGATTTCAGCAAAGG |
| *Tgfb1* | Forward: CTCCCGTGGCTTCTAGTGC |
|  | Reverse: GCCTTAGTTTGGACAGGATCTG |
| *Tgfb2* | Forward: CTTCGACGTGACAGACGCT |
|  | Reverse: GCAGGGGCAGTGTAAACTTATT |
| *Tgfb3* | Forward: CCTGGCCCTGCTGAACTTG |
|  | Reverse: TTGATGTGGCCGAAGTCCAAC |
| *Tgfbr1* | Forward: TCTGCATTGCACTTATGCTGA |
|  | Reverse: AAAGGGCGATCTAGTGATGGA |
| *Tgfbr2* | Forward: CCGCTGCATATCGTCCTGTG |
|  | Reverse: AGTGGATGGATGGTCCTATTACA |
| *Smad1* | Forward: GCTTCGTGAAGGGTTGGGG |
|  | Reverse: CGGATGAAATAGGATTGTGGGG |
| *Smad2* | Forward: ATGTCGTCCATCTTGCCATTC |
|  | Reverse: AACCGTCCTGTTTTCTTTAGCTT |
| *Smad3* | Forward: CACGCAGAACGTGAACACC |
|  | Reverse: GGCAGTAGATAACGTGAGGGA |
| *Smad4* | Forward: AGCCGTCCTTACCCACTGAA |
|  | Reverse: GGTGGTAGTGCTGTTATGATGGT |
| *Smad5* | Forward: TTGTTCAGAGTAGGAACTGCAAC |
|  | Reverse: GAAGCTGAGCAAACTCCTGAT |
| *Smad6* | Forward: TCCGAAGTCCGCTCGGTAG |
|  | Reverse: TCACCGTCTCGCAGTCACT |
| *Smad7* | Forward: GGCCGGATCTCAGGCATTC |
|  | Reverse: TTGGGTATCTGGAGTAAGGAGG |
| *Chop/Ddit3* | Forward: CCTAGCTTGGCTGACAGAG |
|  | Reverse: GTCAGGCGGTCGATTTCC |
| *Actb* | Forward: CTACAGCTTCACCACCACAG |
|  | Reverse: CTACAGCTTCACCACCACAG |
